# Evaluation of Different Drying Methods on the Quality Parameters of *Acanthopanax senticosus* Fruits

**DOI:** 10.3390/foods14071100

**Published:** 2025-03-22

**Authors:** Chunbo Zhao, Zhiqiang He, Xiaoqian Song, Xiaoning Zhang, Yu Xiao, Jia Yu, Minghui Yang, Zhonghua Tang

**Affiliations:** 1 College of Chemistry, Chemical Engineering and Resource Utilization, Northeast Forestry University, Harbin 150040, China; zhaochunbo1992@nefu.edu.cn (C.Z.); hezhiqiang0536@126.com (Z.H.); sxq_824096061@163.com (X.S.);; 2 Key Laboratory of Forest Plant Ecology, Ministry of Education, Northeast Forestry University, Harbin 150040, China; 3 Heilongjiang Provincial Institute of Drug Inspection and Research, Harbin 150088, China

**Keywords:** *Acanthopanax senticosus* (Rupr. & Maxim.), bioactive compounds, antioxidant activity, volatile compounds, functional foods, byproduct

## Abstract

*Acanthopanax senticosus* (Rupr. and Maxim.; AS) fruit, an underutilized byproduct, possesses significant bioactive potential, yet its quality is highly influenced by drying methods. This study systematically evaluated the effects of five drying techniques, including vacuum freeze-drying (VFD), vacuum drying (VD), microwave drying (MD), hot-air drying (HD), and natural drying (ND), on the color retention, bioactive composition, volatile profile, and antioxidant activity of AS fruit. VFD preserved the highest levels of total phenolic content (TPC) and total flavonoid content (TFC), particularly chlorogenic acid, rutin, and quercitrin, leading to superior antioxidant activity. Amino acid analysis indicated that VFD retained the highest concentrations of key essential amino acids, minimizing thermal degradation. Correlation analysis revealed a strong association between TPC, TFC, and antioxidant activity, emphasizing their functional significance. Through multivariate statistical analysis, 12 volatile compounds were identified as potential biomarkers to distinguish AS fruit samples processed using different drying methods, highlighting significant metabolic differences between drying techniques. Overall, VFD emerged as the optimal method for preserving AS fruit’s bioactive integrity, offering valuable insights for post-harvest processing strategies in the nutraceutical industry.

## 1. Introduction

*Acanthopanax senticosus* (Rupr. and Maxim.), commonly known as Siberian ginseng, is native to the northern and eastern regions of China and is also found in parts of Korea, Japan, and Russia’s Far East [1,2]. As a medicinal and edible plant, AS has been widely used in traditional Chinese medicine and functional foods. The roots and rhizomes of AS, officially recognized in various pharmacopeias, are renowned for their therapeutic properties, including tonifying the kidney and nourishing essence, strengthening muscles and bones, enhancing cognitive function, and improving physical endurance with prolonged use [3,4,5].

Despite the extensive utilization of AS, its fruits are often discarded as low-value agricultural byproducts during harvesting and processing, leading to significant resource wastage and environmental concerns. However, recent studies have demonstrated that AS fruits possess comparable biological and pharmacological value to their traditionally used parts, making them a promising candidate for functional food development [6,7]. Rich in flavonoids, saponins, polysaccharides, and volatile compounds [8], AS fruits exhibit notable antioxidant [9], anti-inflammatory [10], anti-tumor [11], anti-fatigue effects [12], and immunomodulatory properties [13]. Nevertheless, the quality, bioactive composition, and physicochemical properties of AS fruits are highly susceptible to post-harvest processing methods, particularly drying techniques, which can significantly impact their chemical stability and overall product quality.

Drying is a crucial post-harvest processing step for medicinal and functional fruits, as it directly influences the preservation of bioactive compounds, the retention of volatile components, and therapeutic properties of the final product [14]. Various drying techniques, including HD, VFD, and MD, induce distinct physicochemical changes that can alter the physicochemical properties, bioactive constituents, aroma profile, and bioactivity of AS fruits [15,16,17]. Among these factors, volatile compounds play a key role in defining the fruit’s aromatic characteristics, yet they are highly prone to thermal degradation and oxidative deterioration. Thus, a comprehensive evaluation of how different drying techniques influence the volatile composition and metabolic profile of AS fruits is essential for optimizing post-harvest processing strategies.

This study aims to systematically compare the effects of five different drying methods (VFD, VD, MD, HD, and ND) on the quality attributes, bioactive compounds, and volatile components of AS fruit. By analyzing color parameters, volatile profiles, bioactive constituents and functions, this research seeks to identify the most effective drying technique for preserving the fruit’s physicochemical and functional properties. The findings will provide valuable insights for optimizing post-harvest processing methods, ensuring product quality, and supporting the commercial utilization of AS fruit in the nutraceutical industry.

## 2. Materials and Methods

### 2.1. Preparation of Materials

**Figure 1:**
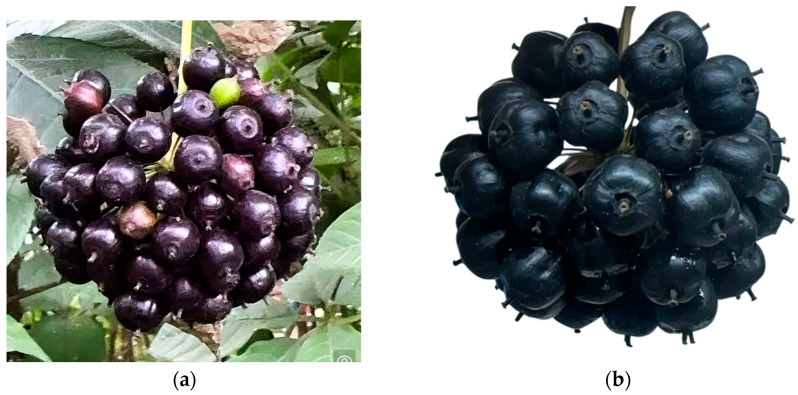
Morphological characteristics of AS fruits at different stages: (**a**) AS fruits during the fruiting period; (**b**) freshly harvested AS fruits.

The AS fruits were harvested at the commercial maturity stage, characterized by the deep purplish-black color and firm texture shown in Figure 1. The maturity (BBCH 93) of the harvested fruits was determined based on both manual experience and the Biologische Bundesanstalt, Bundessortenamt und Chemische Industrie (BBCH) scale. All AS fruits were collected from the garden located at 126°38′17.268″ E, 45°43′26.684″ N, within the premises of the College of Chemical Engineering and Resource Utilization, Northeast Forestry University.

After harvesting, the fruits were promptly transported to the laboratory within 1 h. Only intact and fresh fruits were selected and mixed together to minimize the impact of sample heterogeneity. All samples were rinsed with purified water at 20 ± 2 °C to remove surface impurities. No detergents or chemical cleaning agents were used. The rinsed fruits were gently blotted with sterile cotton fabric to remove surface moisture. They were then spread in a single layer on perforated stainless-steel trays and left at room temperature with natural air circulation for 1 h before different drying treatments (VFD, HD, VD, MD, and ND).

### 2.2. Drying and Sample Extraction

VFD samples were pre-frozen at −80 °C for 12 h (DW-86-386, Haier Biomedical Co., Ltd., Qingdao, China) to ensure uniform solidification. Lyophilization was conducted at −20 °C under a pressure of 7.3 Pa for 48 h, with the condenser temperature maintained at −88.7 °C (FB-1B-80+, Bo Yikang Instruments Co., Beijing, China). For HD, the samples were dried at 40 °C for 48 h in a forced-air drying oven (DHG-9245A, Shanghai Yiheng Technology Co., Shanghai, China), with air circulation at 1200 rpm and 15 air exchanges per hour. VD was executed in a vacuum chamber (VO29, Memmert GmbH + Co., KG, Schwabach, Germany) at 40 °C under a pressure of 20 Pa for 16 h. For MD, the operation was carried out in a microwave oven (XMJD6SW-2, Xiaomi Mechanical Instrument Co., Ltd., Nanjing, China) for a duration of 15 min, with a microwave power of 700 W and a microwave frequency of 2.45 GHz. ND samples were spread uniformly on perforated stainless-steel trays and dried naturally at ambient temperature (20–25 °C) for 25 days. Overnight, trays were transferred to an incubator (DHP-9082, Shanghai Jinghong Experimental Equipment Co., Shanghai, China) maintained at 23 ± 1 °C and 45 ± 5% RH. Subsequently, all dried samples were ground at 25 ± 2 °C using a high-speed blender (FW100, Taisite Instrument Co., Ltd., Tianjin, China) in five cycles (10 s grinding with 30 s cooling intervals). Powders were sieved (3.35 mm), vacuum-packed (−0.08 MPa) using a vacuum packaging machine (DZ-260PD, Hualian Machinery, China), sealed in low-density polyethylene bottles, and stored at −80 °C until analysis.

Regarding the extraction process of the dried fruit powder, we employed an ultrasound-assisted extraction method. Specifically, approximately 1 g of the sample was accurately weighed and diluted to a final volume of 50 mL with 70% (*v/v*) aqueous ethanol. The mixture was then subjected to ultrasonic treatment at 300 W for 1 h and subsequently stored at 4 °C for further use.

### 2.3. Determination of Color Properties and Moisture Content

The color parameters of AS samples were evaluated by employing a SY-1 colorimeter (Tianjin Tianda Tianfa Technology Co., Ltd., Tianjin, China). In the color measurement system, the L* value stands for the degree of lightness or darkness, the a* value represents the degree of redness versus greenness, and the b* value corresponds to the degree of yellowness compared to blueness. The total color difference (ΔE), which reflects the extent of overall color changes in the samples, was calculated in accordance with Equation (1).

(1) $$\Delta E=\sqrt[{2}]{{\left(L^{*}-L_{0}^{*}\right)}^{2}+{\left(a^{*}-a_{0}^{*}\right)}^{2}+{\left(b^{*}-b_{0}^{*}\right)}^{2}}$$

ΔE: The total color difference;

L*: Lightness value;

a*: Chromaticity coordinate on the red-green axis;

b*: Chromaticity coordinate on the yellow-blue axis;

L_0_*, a_0_*, and b_0_*: The corresponding L*, a*, and b* values of the reference sample (fresh fruit).

Moisture content of dried AS fruits was determined by volumetric Karl Fischer titration using a fully automated titrator (Metrohm 915 KF Ti-Touch, Metrohm AG, Herisau, Switzerland). Approximately 1 g of powdered sample was dispersed uniformly in anhydrous methanol (Sigma-Aldrich, St. Louis, MI, USA) under continuous stirring to facilitate moisture extraction. Prior to analysis, instrument calibration was performed using a standard water solution (Merck KGaA, Darmstadt, Germany), and environmental moisture interference was eliminated by blank titration. The volumetric Karl Fischer reagent (water equivalence: 5.00 mg H_2_O/mL, Merck KGaA, Darmstadt, Germany) was titrated until a stable endpoint was reached, and titrant consumption (mL) was recorded. The moisture content (MC, %) was calculated using the formula:

(2) MC (%)=(F×V)/W×100

F: The water equivalence of the titrant (5.00 mg H_2_O/mL);

W: Sample weight (mg);

V: The consumed titrant volume (mL).

### 2.4. Determination of Total Polyphenols and Flavonoids

TPC (total phenolic content) was determined using a modified microplate Folin–Ciocalteu reagent method [18]. Briefly, 20 μL sample extracted in Section 2.1 was mixed with 20 μL Folin–Ciocalteu reagent (Sigma-Aldrich, St. Louis, USA), shaken (medium speed) for 2 min using a microplate reader (SpectraMax^®^ iD3, Molecular Devices, San Jose, CA, USA), and left undisturbed for 6 min. Subsequently, 200 μL Na_2_CO_3_ solution (7%, *w/v*; Sinopharm Chemical Reagent Co., Ltd., Shanghai, China) and 10 μL purified water were added, followed by shaking for 1 min. After incubation for 120 min in the dark (25 ± 2 °C), absorbance was measured at 750 nm. Results were expressed as mg gallic acid equivalents (mg GAE)/g based on a gallic acid standard curve (Shanghai Yuanye Bio-Technology, Shanghai, China).

TFC (total flavonoid content) is determined based on the reaction with the aluminum trichloride (AlCl_3_) reagent method [19]. Briefly, 50 μL of sample extracted in Section 2.1 was mixed with 100 μL methanol (Honeywell Research Chemicals, Morris Plains, NJ, USA) and 20 μL of 10% (*w/v*) AlCl_3_ solution (Sinopharm Chemical Reagent Co., Ltd., Shanghai, China). After gentle mixing, the mixture was incubated at room temperature (25 ± 2 °C) for 3 min. Subsequently, 20 μL of 1 M CH_3_COONa solution (Sinopharm Chemical Reagent Co., Ltd., Shanghai, China) and 60 μL methanol were sequentially added, followed by incubation for 40 min in dark (25 ± 2 °C). Absorbance was measured at 430 nm. TFC was expressed as mg quercetin equivalents (mg QE)/g based on a quercetin standard curve (Shanghai Yuanye Bio-Technology, Shanghai, China).

### 2.5. Determination of Amino Acids

Amino acids were analyzed using an LA8080 amino acid analyzer (Hitachi High-Tech Corporation, Tokyo, Japan). Briefly, a 0.5 g sample was extracted with 30 mL anhydrous ethanol via vigorous vortexing (2 min), diluted to 50 mL with purified water (conductivity ≤ 0.055 μS/cm), frozen at −20 °C for 30 min, and centrifuged (11,180× *g*, 5 min, 4 °C). The supernatant (1 mL) was extracted using a micropipette (Eppendorf Research^®^ Plus, Hamburg, Germany) and filtered through a 0.22 μm Polyvinylidene Fluoride (PVDF) syringe filter before analysis. Amino acids were separated using a sulfonic acid-type cation exchange resin column and detected at 570 and 440 nm. Aspartic acid (Asp), threonine (Thr), serine (Ser), glutamic acid (Glu), glycine (Gly), alanine (Ala), cysteine (Cys), valine (Val), methionine (Met), isoleucine (Ile), leucine (Leu), tyrosine (Tyr), phenylalanine (Phe), lysine (Lys), histidine (His), arginine (Arg), and proline (Pro) were quantified based on peak area using external standards (mixed amino acid standard solution, 2.5 μmol/mL, Tianjin Alta Science, Tianjin, China).

### 2.6. Quantitative Analysis of the Main Bioactive Substances

Eight bioactive compounds (eleutheroside B and E, protocatechuic acid, chlorogenic acid, rutin, hyperoside, isofraxidin, and quercitrin) were simultaneously quantified using ultra-performance liquid chromatography (UPLC-UV, Waters Corporation, Milford, MA, USA). Standards were obtained from Shanghai Yuanye Bio-Technology Co., Ltd. (Shanghai, China) Sample extraction followed a modified method [20]. A 1 g sample was extracted with 30 mL ethanol–water (80:20, *v/v*) for 1 h (25 ± 2 °C), centrifuged (1880× *g*, 4 °C, 15 min), and filtered through a 0.22 µm PVDF membrane. Chromatographic separation was achieved on an ACQUITY UPLC^®^ BEH C18 column (100 mm × 2.1 mm, 1.7 μm, Waters Corporation, Milford, MA, USA) at 27 °C, using gradient elution (Table S1) with mobile phases consisting of solvent A (acetonitrile–phosphoric acid, 980:20, *v/v*) and solvent B (acetonitrile) at 0.3 mL/min. Detection was performed at 210 nm. Standard solutions and sample extracts were injected separately, with quantification based on external standard calibration curves.

### 2.7. Volatile Compound Analyses

Volatile compounds were analyzed using headspace solid-phase microextraction coupled with gas chromatography–mass spectrometry (HS-SPME-GC-MS), following a modified protocol [21]. Briefly, 1.0 g of sample and the internal standard (1,2-dichlorobenzene, 100 ng/μL, 4 μL) were placed into a 20 mL headspace vial. After equilibration at 100 °C for 5 min, volatile compounds were extracted using a PDMS/DVB/CAR-coated fiber at 100 °C for 15 min, followed by thermal desorption at 250 °C for 5 min. The GC-MS system (Agilent 7000C, Agilent Technologies, Santa Clara, CA, USA) equipped with a DB-5MS column (30 m × 0.25 mm × 0.25 μm, GL Sciences Inc., Tokyo, Japan) utilized helium (1.0 mL/min) in splitless injection mode. The oven temperature was programmed as follows: held at 40 °C (3.5 min), increased to 100 °C at 10 °C/min, then to 180 °C at 7 °C/min, and finally to 280 °C at 25 °C/min, maintained for 5 min. Electron ionization (EI) was set at 230 °C, with mass scans ranging from 35 to 550 *m*/*z*. Compound identification was based on mass spectral matching with the National Institute of Standards and Technology Library 14 (NIST 14) (similarity ≥ 70%), retention index (RI) comparison with literature values, and the mass-to-charge (*m*/*z*) ratios of characteristic fragment ions.

### 2.8. Determination of the Antioxidant Activity

#### 2.8.1. DPPH Radical Scavenging Capacity

1,1-diphenyl-2-picryl hydrazyl (DPPH) radical scavenging activity was evaluated following a modified method [22]. Briefly, 40 μL of sample extract (Section 2.1) was mixed with 160 μL purified water and 40 μL freshly prepared DPPH solution (Sigma-Aldrich, St. Louis, MO, USA). After incubation in darkness at 25 °C for 30 min, absorbance was measured at 517 nm using a microplate reader (SpectraMax^®^ iD3, San Jose, CA, USA). A control was prepared identically, substituting the sample with purified water. The radical scavenging activity (%) was calculated using the following equation:

DPPH radical scavenging activity (%) = [1 − (A_sample_/A_blank_)] × 100 (3)

#### 2.8.2. ABTS Radical Scavenging Capacity

The 2,2’-Azinobis-(3-ethylbenzthiazoline-6-sulphonate) (ABTS) assay was conducted following the modified method [23]. Briefly, ABTS radical stock solution was prepared by mixing 7.4 mM ABTS (Sigma-Aldrich, St. Louis, MO, USA) with 2.6 mM potassium persulfate (Sinopharm Chemical Reagent Co., Ltd., Shanghai, China) both in purified water, and incubated in darkness at 25 °C for 12 h. The solution was diluted with 80% (*v/v*) aqueous methanol to achieve an absorbance of 1.10 ± 0.02 at 734 nm. Subsequently, 15 µL sample extract (Section 2.1) was mixed with 285 µL diluted ABTS solution, incubated in darkness at 25 °C for 2 h, and absorbance measured at 734 nm. A blank was prepared identically using purified water instead of the sample. ABTS radical scavenging activity (%) was calculated using the equation:

ABTS radical scavenging activity (%) = [1 − (A_sample_/A_blank_)] × 100 (4)

### 2.9. Statistical Analyses

All assays were performed in triplicate, and results are expressed as mean ± standard deviation (SD). Statistical analyses were conducted using SPSS Statistics 27 (IBM, Chicago, IL, USA). One-way analysis of variance (ANOVA), Tukey test, and Duncan’s multiple range test were employed at a 95% confidence level. Principal component analysis (PCA), partial least squares discriminant analysis (PLS-DA), and Orthogonal Partial Least Squares Discriminant Analysis (OPLS-DA) were performed using SIMCA 14.1 (Umetrics, Umeå, Sweden). Histograms and volcano plots were generated using OriginPro 2021 (OriginLab Corp., Northampton, MA, USA).

## 3. Results

### 3.1. Color Properties

Color is a key quality indicator influencing consumer acceptability of food products [24]. Different drying methods result in variations in color, typically assessed using L*, a*, and ΔE. The results indicate significant differences in these parameters across drying methods, as shown in Table 1.

VFD-treated samples exhibited the highest L* value (33.56 ± 0.23) and the lowest ΔE (1.88 ± 0.33), indicating superior color preservation. The sublimation process under low temperature and pressure minimizes enzymatic oxidation and pigment degradation, effectively preventing browning. The low a (0.52 ± 0.02) and b (−3.33 ± 0.21) values further confirm VFD’s effectiveness in preventing color deterioration. Studies have demonstrated that freeze-drying retains bioactive compounds and reduces color changes more effectively than other drying methods [25,26]. HD-treated samples had the lowest L* value (22.66 ± 0.56) and the highest ΔE (11.11 ± 0.50), indicating significant darkening and color degradation. Xu et al. (2020) reported that hot-air drying caused a substantial decrease in L* and an increase in ΔE, confirming severe color deterioration [27].

ND-treated samples maintained a relatively high a value (3.17 ± 0.15), suggesting that low-temperature air drying preserved anthocyanins to some extent. However, an ∆E value of 6.77 ± 0.26 indicates moderate color changes due to prolonged drying exposure. VD-treated samples exhibited a relatively high L* (30.36 ± 0.26) and a low ΔE (2.53 ± 0.20), reflecting the protective effects of vacuum conditions in reducing oxidative degradation. MD-treated samples showed moderate L* (27.41 ± 0.22) and an intermediate ΔE (6.14 ± 0.24), indicating that rapid internal heating limited excessive color loss but still caused some pigment degradation. MD-treated samples exhibited moderate L* (27.41 ± 0.22) and an intermediate ΔE (6.14 ± 0.24), indicating that rapid internal heating prevents excessive color loss but still induces some pigment degradation. The moderate a value (2.18 ± 0.14)* and b value (−6.38 ± 0.05)* suggest that some anthocyanin loss and browning reactions occurred during microwave processing.

Drying methods not only affect fruit moisture content but also play a crucial role in the retention of bioactive compounds and antioxidant activity. ND-treated samples retained the highest moisture content (14.97 ± 0.12%), consistent with previous findings [28]. While ND reduces thermal degradation, prolonged exposure to air and oxygen leads to polyphenol and vitamin C degradation [29]. HD (9.48 ± 0.02%) and VD (8.54 ± 0.12%) showed moderate water removal efficiency, but their effects on bioactive compound retention varied significantly. High temperatures accelerate moisture evaporation but also degrade antioxidants [30]. Vacuum drying enables lower-temperature dehydration, reducing thermal degradation, although its water removal efficiency is lower than MD and VFD. MD (6.63 ± 0.18%) and VFD (5.91 ± 0.14%) achieved the most effective dehydration while preserving the highest levels of bioactive compounds.

### 3.2. Determination of TPC and TFC

As shown in Table S2, drying methods significantly affected TFC and TPC levels in AS fruits. VFD (131.9 mg/g) and ND (125.5 mg/g) retained the highest TFC without significant differences, followed by moderate retention in MD (90.0 mg/g) and HD (78.3 mg/g). VD (11.4 mg/g) exhibited the lowest TFC, highlighting its limited suitability for preserving heat-sensitive flavonoids.

The superior flavonoid retention in VFD is attributed to its low-temperature process, which stabilizes tissue structure and minimizes flavonoid loss. Similarly, ND avoids high temperatures, preventing oxidative degradation. Flavonoids, containing phenolic hydroxyl groups, are highly susceptible to heat-induced oxidation [31], explaining the significant TFC reduction in HD and MD. VD’s rapid heating and vacuum conditions likely accelerate degradation, resulting in minimal retention. This aligns with Albanese, D et al., 2013, who found that vacuum freeze-drying preserved higher TPC and antioxidant capacity in mulberry fruits compared to vacuum drying, highlighting the role of oxygen restriction in bioactive compound preservation [32].

TPC followed a similar trend. VFD (23.62 ± 1.57 mg/g) achieved the highest retention, followed by ND (19.60 ± 1.22 mg/g), MD (15.26 ± 3.36 mg/g), and HD (8.93 ± 1.79 mg/g), with VG (0.93 ± 0.11 mg/g) showing the lowest value. VFD further enhances TPC retention by restricting oxygen exposure during the drying process, significantly reducing oxidation. ND’s low-temperature, gradual drying minimizes oxidative degradation, preserving phenolic compounds and maintaining cell structure integrity.

In contrast, HD and MD promote degradation due to higher temperatures, while VG’s extreme vacuum and thermal stress result in severe phenolic loss. Tan, S et al. (2021) similarly reported significant TPC and antioxidant declines in Rhodomyrtus tomentosa berries following microwave and hot-air drying [33]. Zhao, G et al. (2017) found that drying generally reduces TPC and antioxidant activity in fruits, with ambient drying causing greater losses than oven drying, highlighting the importance of controlled drying conditions for bioactive compound preservation [34].

### 3.3. Quantitative Analysis of Main Active Ingredients

The retention of bioactive compounds is critical for the functional properties of AS fruit. This study evaluated the impact of five drying methods on phenolic acids, flavonoids, and eleutherosides (Table 2). Method validation results are summarized in Table S3. Representative chromatograms of standards and samples are shown in Figures S1 and S2. VFD exhibited superior preservation of key phenolic compounds, including chlorogenic acid (161.66 mg/100 g), rutin (601.20 mg/100 g), and quercitrin (96.64 mg/100 g). In contrast, HD retained higher levels of eleutheroside E, isofraxidin, and protocatechuic acid. VD, despite reduced pressure, resulted in substantial losses of protocatechuic acid, chlorogenic acid, rutin, hyperoside, and quercitrin. These findings highlight the significant influence of drying methods on the stability and degradation of phenolic and glycoside compounds in AS fruits. The enhanced retention of phenolics in VFD-treated samples is attributed to its low-temperature, low-pressure conditions, which effectively mitigate polyphenol degradation.

Among AS fruit bioactive compounds, eleutheroside B exhibited the highest content in VFD (161.66 mg/100 g), significantly exceeding levels observed with other drying methods. According to the Arrhenius equation, reaction rates increase exponentially with temperature [35]. In VFD, extremely low temperatures minimize reaction rate constants, significantly slowing chemical degradation. Under standard conditions, the oxidation and decomposition of eleutheroside B require sufficient activation energy. However, in VFD, energy input remains below this threshold, preserving structural integrity. Additionally, glycosides such as eleutheroside E and isofraxidin undergo hydrolysis at elevated temperatures due to glycosidic bond cleavage. The low-temperature environment of VFD prevents hydrolysis, enhancing glycoside retention and stability. Furthermore, VFD’s high-vacuum conditions limit oxygen exposure, effectively reducing oxidation-induced phenolic degradation.

The highest levels of eleutheroside E (20.48 mg/100 g) and isofraxidin (2.32 mg/100 g) were observed in HD-treated samples. At high temperatures, eleutheroside E may undergo thermal decomposition; however, HD’s rapid moisture removal limits hydrolysis, maintaining its stability. In contrast, VFD’s low-temperature environment slows chemical reactions, preventing hydrolysis and oxidation, thereby preserving glycosidic bonds. Isofraxidin, a coumarin compound, exhibits thermal stability due to its conjugated system, which mitigates oxidative stress and resists enzymatic degradation. Under HD conditions, it remains relatively stable, as it is not a primary substrate for enzymatic reactions in fruits. Although VFD retained lower levels of eleutheroside E and isofraxidin than HD, it outperformed other drying methods. VD, conducted under low pressure, resulted in poor bioactive compound retention, likely due to prolonged drying times. ND, a slow process with extended light and humidity exposure, led to significant losses of key compounds such as rutin and hyperoside. Previous research indicated that hot-air flow rolling dry-blanching pretreatment significantly improved drying efficiency and enhanced phenolic and flavonoid retention by inhibiting oxidative enzymes in AS fruits [36]. Similarly, freeze-drying demonstrated superior retention of total phenolics and beta-carotene in African eggplant, confirming the advantage of low-temperature drying for preserving bioactive components [37].

### 3.4. Principal Component Analysis of Main Active Ingredients

PCA, a multivariate statistical technique, reduces data dimensionality while preserving key variance, enabling pattern recognition and sample differentiation [38,39]. To assess the impact of drying methods on AS fruit bioactive compounds, PCA was conducted (Figure S3) using quantified compounds: protocatechuic acid, chlorogenic acid, rutin, hyperoside, isofraxidin, quercitrin, eleutheroside B, and eleutheroside E. The first two principal components (PC1: 58.0%, PC2: 25.1%) explained a cumulative 83.1% variance, demonstrating distinct compositional differences among drying methods.

VFD samples were positioned on the far right of PC1, indicating the most distinct characteristics among all drying methods. As PC1 integrates multiple variables, it reflects differences in volatile retention and chemical integrity, both of which are better preserved under VFD’s low-temperature, low-pressure conditions. The high PC1 score of VFD suggests superior retention of key bioactive compounds, particularly phenolics. Similar findings were reported in dried Omija fruits, where PCA effectively differentiated drying effects on volatile and bioactive compounds [40].

### 3.5. Analysis of Amino Acid Composition

Amino acids are crucial for plant metabolism, serving as precursors for proteins, secondary metabolites, and stress-responsive compounds [41,42]. Their composition is significantly influenced by external factors, including drying methods [43]. As shown in Table 3, 17 amino acids were identified in AS fruits across different drying treatments, with total content ranging from 3.934 to 11.590 mg/g. Arg was the most abundant, contributing 27.1–31.1% of total free amino acids, with concentrations varying from 1.070 to 3.610 mg/g. VFD retained the highest Arg content (3.610 mg/g), followed by VD (2.900 mg/g), HD (2.070 mg/g), and MD (1.070 mg/g). Notably, Arg levels in VFD samples were approximately 3.4-times higher than in MD samples, highlighting the superior preservation of amino acids under low-temperature drying conditions.

VFD preserved the highest levels of Asp, Thr, Ser, Glu, Cys, Met, Tyr, His, and Arg, whereas VD retained higher concentrations of Gly, Val, Ile, Leu, Phe, and Lys. HD was most effective in preserving Ala, while ND resulted in the lowest levels of Asp, Thr, Ser, Gly, Leu, Phe, and Tyr. High-temperature drying methods, such as HD, may induce thermal degradation of heat-sensitive amino acids like methionine and glutamic acid, primarily due to the Maillard reaction. This reaction, which accelerates at elevated temperatures, leads to interactions between amino acids and reducing sugars, causing structural modifications and reduced amino acid content [44]. In contrast, VFD’s low-temperature conditions effectively mitigate thermal degradation, preserving amino acid integrity [44].

The vacuum environment in VD may alter the redox state of amino acids, potentially affecting substance transfer and reaction dynamics, as indicated by the higher arginine content. In contrast, MD’s electromagnetic fields can disrupt molecular structures and weaken the chemical bond stability of amino acids. Microwave heating can induce protein denaturation, leading to the release of amino acids. However, excessive microwave heating may result in the degradation of amino acids, particularly heat-sensitive ones such as lysine.

### 3.6. Volatile Compounds Evaluation

#### 3.6.1. Preliminary Identification of Volatile Compounds

A total of 60 volatile compounds were identified via mass spectral matching (NIST 14), retention indices (RIs), and characteristic *m*/*z* fragment ions (Table S4). These included 48 hydrocarbons (80.0%), 4 alcohols (6.7%), 5 ketones (8.3%), 2 esters (3.1%), and 1 aldehyde (1.7%), with terpenes comprising 89.6% of all hydrocarbons. The number of identified compounds varied across drying methods: ND (58), HD (56), VFD (54), VD (54), and MD (48).

**Figure 2:**
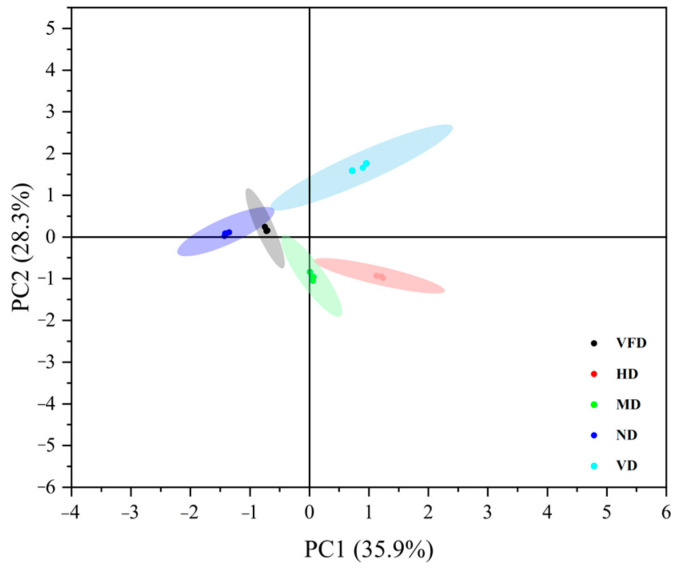
Principal component analysis (PCA) score plot of volatile compounds in AS fruits under different drying methods (*n* = 3).

**Figure 3:**
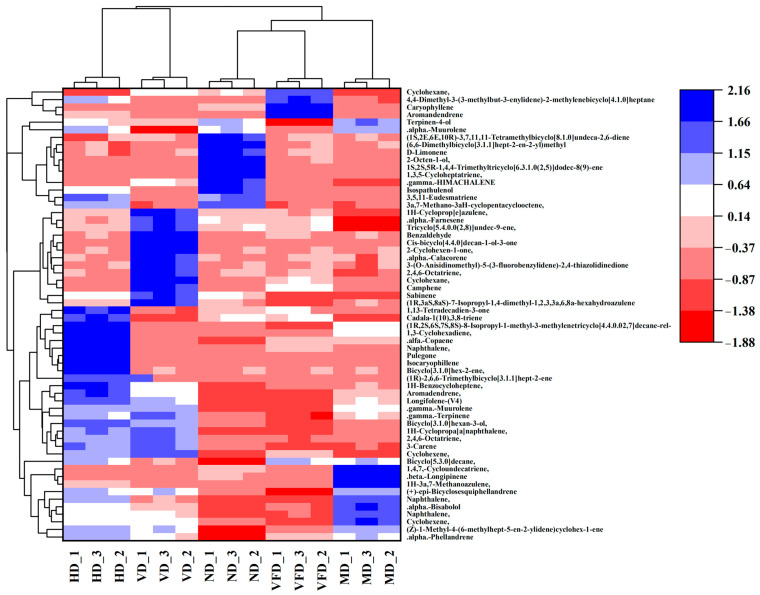
The heatmap of volatile compounds in AS fruits under different drying methods (*n* = 3).

PCA and HCA confirmed significant differences in volatile composition (Figure 2). Both results, based on the normalized relative peak areas of 60 volatile compounds (*n* = 3), explained 64.2% of the variance (PC1: 35.9%, PC2: 28.3%), with ND and VFD forming distinct clusters, indicating effective volatile compound retention. HCA (Figure 3) further validated this clustering, grouping ND and VFD together, while HD and MD clustered separately, reflecting the greater impact of high-temperature drying on volatile degradation. These findings underscore the importance of optimizing drying strategies to preserve the aroma and flavor profile of AS fruits. A study reported a 59% decrease in total volatiles in dried mangoes, particularly monoterpenes and sesquiterpenes, due to thermal degradation, oxidation, and evaporation [45].

#### 3.6.2. The Contribution of Volatile Compounds

To assess the impact of drying methods on volatile profiles, PLS-DA was conducted using SIMCA 14.1 (Figure S4), with normalized relative peak areas (%) of 60 identified volatile compounds as input variables. The model demonstrated strong explanatory and predictive capabilities. The PLS-DA score plot showed that VFD and ND clustered closely, indicating similar volatile retention, while HD, VD, and MD were distinctly separated, consistent with PCA results.

To evaluate the contribution of individual volatile compounds to group differentiation, Variable Importance in Projection (VIP) scores were calculated using PLS-DA, with VIP > 1 and *p* < 0.05 considered significant [46,47]. As shown in Figure S5a, 28 compounds had VIP > 1, indicating their substantial role in distinguishing drying methods (compound details in Table S5). Further statistical analysis identified 12 key volatiles, meeting both VIP > 1 and *p* < 0.05 criteria, confirming their significance in sample classification (Table S6). These compounds could serve as potential biomarkers for differentiating AS fruit samples processed by various drying techniques. To assess potential overfitting, 200 rounds of cross-validation were performed, evaluating the R^2^ and Q^2^ values. The validation plot (Figure S5b) displayed a steep slope, confirming that the PLS-DA model was not overfitted.

### 3.7. Effect of Drying Treatments on the Metabolite Profiles of AS Fruit

Different dehydration treatments significantly impact the metabolic composition of AS fruits [48]. Orthogonal Partial Least Squares Discriminant Analysis (OPLS-DA) is a widely used dimensionality reduction technique that effectively identifies key metabolites responsible for group separation [49,50]. Volcano plots further facilitate this analysis by visualizing the statistical significance (*p*-value) against the magnitude of change (fold change) in metabolites, making them particularly useful for detecting biologically relevant variations [51].

**Figure 4:**
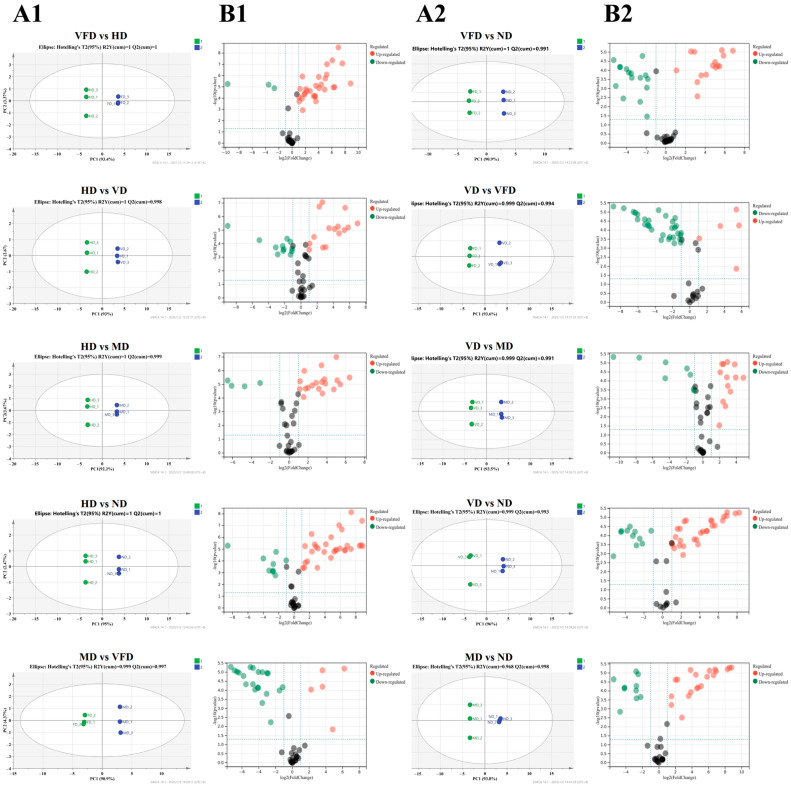
Metabolic profiles of AS fruits subjected to five different drying methods, analyzed using HS-SPME-GC-MS. OPLS-DA score plots illustrate the separation of metabolite profiles among drying treatments (Panel **A1**,**A2**), while volcano plots highlight significant metabolic variations (Panel **B1**,**B2**). The vertical blue dashed lines indicate the fold change (FC) threshold, and the horizontal blue dashed line represents the significance threshold (*p*-value). Black dots denote compounds without significant differential expression.

To evaluate the metabolic differences induced by different drying methods, OPLS-DA models and volcano plots were employed. In OPLS-DA analysis, we used the normalized response values of 60 volatile compounds detected. The result demonstrated distinct metabolic variations among different drying methods. The OPLS-DA score plot revealed two well-separated clusters (R^2^Ycum > 0.9, Q^2^cum > 0.9), indicating strong model reliability and predictive accuracy (Figure 4(A1,A2)) [52]. Additionally, the volcano plots (Figure 4(B1,B2)) demonstrated significant differences in metabolite distribution across the five drying methods, further confirming that drying treatments lead to substantial metabolic alterations. These findings underscore the strong discriminative capability of OPLS-DA and highlight the influence of drying methods on the metabolite composition of AS fruits.

### 3.8. Determination of Antioxidant Activity

Antioxidant capacity is a crucial indicator for evaluating the functional properties of food products [53,54]. As shown in Table S7, significant differences were observed among the groups for both DPPH and ABTS assays (*p ≤* 0.05), indicating that the drying method substantially affects antioxidant retention.

DPPH radical scavenging activity is widely used to assess antioxidant potential, where higher values indicate stronger free radical scavenging capacity [55]. Among all groups, the VFD group exhibited the highest DPPH scavenging activity (65.3 ± 2.6%), significantly higher than the other groups (*p ≤* 0.05), indicating that this method best preserves antioxidant compounds. The ND group (59.7 ± 1.5%) also displayed relatively high DPPH activity, suggesting that the slow drying process may help mitigate the degradation of bioactive compounds. In contrast, HD (40.7 ± 0.7%) and MD (46.8 ± 2.1%) exhibited lower DPPH scavenging capacity, suggesting that high-temperature processing may lead to the degradation or oxidation of antioxidant components. The VD group had the lowest DPPH scavenging activity (11.5 ± 0.1%), significantly lower than all other treatments (*p ≤* 0.05), indicating that this drying method may result in substantial degradation or structural changes of bioactive compounds, leading to a dramatic reduction in antioxidant capacity. Vacuum drying effectively preserved the antioxidant properties of Syzygium caryophyllatum fruit, particularly in retaining DPPH radical scavenging activity, whereas sun drying led to the greatest loss of antioxidant potential [56].

ABTS radical scavenging capacity provides complementary insights into antioxidant potential, often used in combination with the DPPH assay for a more comprehensive evaluation [57]. The overall trends observed in the ABTS assay were consistent with the DPPH results. The VFD group showed the highest ABTS scavenging activity (32.1 ± 0.6%), significantly superior to all other drying methods (*p ≤* 0.05), further confirming the effectiveness of freeze-drying in preserving antioxidant activity. The ND (25.1 ± 1.1%) and MD (21.6 ± 0.7%) groups exhibited moderate ABTS radical scavenging activity, suggesting that these methods retain antioxidant compounds to some extent but still induce some level of degradation. Similar to the DPPH results, the HD (14.6 ± 0.3%) and VD (4.3 ± 0.1%) groups had the lowest ABTS scavenging activity, highlighting that these drying methods may cause severe oxidative damage or degradation of antioxidant compounds. The effect of different drying methods on mango, avocado, and tomato showed that freeze-drying retained the highest levels of antioxidants compared to refractance window and oven drying methods [58].

### 3.9. Correlation Analysis of Physicochemical Properties, Bioactive Compounds, and Antioxidant Activity

**Figure 5:**
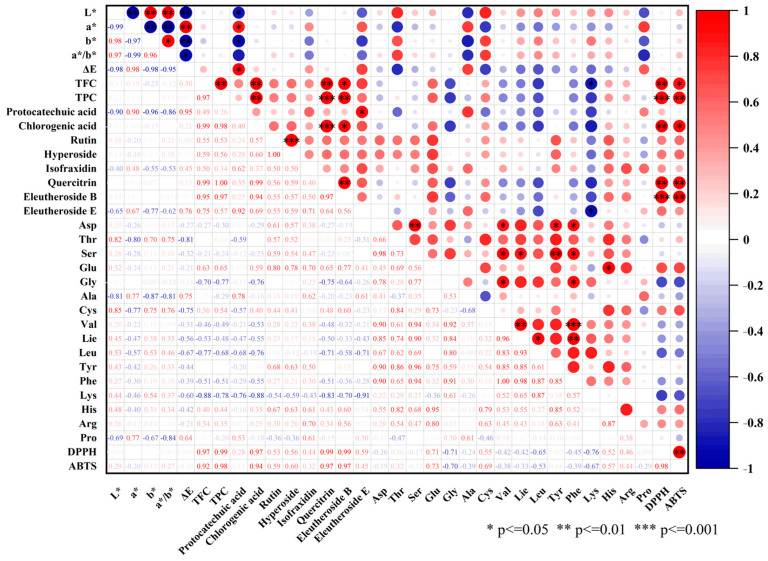
Correlation heatmap illustrating Pearson correlation coefficients among quality parameters, bioactive constituents, and antioxidant activities in AS fruit samples subjected to different drying methods.

Correlation analysis is a powerful statistical tool used to evaluate the strength and direction of relationships between multiple variables, providing insights into potential associations and underlying mechanisms [59,60]. The correlation heatmap (Figure 5) provides a comprehensive overview of the relationships among color parameters, TFC, TPC, bioactive compounds, amino acids, and antioxidant activities (DPPH and ABTS radical scavenging assays).

#### 3.9.1. Color Parameters

As shown in Figure 5, L* was strongly positively correlated with b and a*/b* (r = 0.98, *p* < 0.001), suggesting that brighter samples retained more yellow pigments, possibly due to lower carotenoid degradation. A similar trend has been validated in other studies. For example, Turkiewicz et al. (2019) investigated the drying process of Japanese quince and found that freeze-drying effectively reduced carotenoid degradation, maintaining a higher level of yellow pigments [44]. Conversely, L* was negatively correlated with a* (r = −0.99, *p* < 0.001), indicating that increased brightness was associated with reduced redness, likely due to anthocyanin degradation or lower Maillard reaction intensity. This result is consistent with the finding on the blueberry drying process, which demonstrated that freeze-dried samples exhibited a higher anthocyanin retention rate and a lower reduction in redness compared to hot-air-dried samples [61].

a* was negatively correlated with b and a*/b* (r = −0.97, *p* < 0.01), meaning redder samples exhibited reduced yellowness, likely due to polyphenol oxidation. A similar trend was also observed in a study by Turkmen et al. (2020), which reported that different drying methods had a significant impact on the color parameters of cherry laurel fruit. Specifically, freeze-drying was found to better preserve the red hue, whereas hot-air drying was more prone to anthocyanin degradation [62]. Furthermore, a was strongly and positively correlated with ΔE (r = 0.99, *p* < 0.01) and protocatechuic acid (r = 0.90, *p ≤* 0.05), indicating that samples undergoing greater color changes exhibit increased red intensity and higher levels of protocatechuic acid. This finding suggests that drying-induced oxidation or polymerization of phenolic compounds plays a critical role in color development.

b* was negatively correlated with ΔE (r = −0.98, *p ≤* 0.05) and protocatechuic acid (r = −0.96, *p ≤* 0.05), indicating that greater color shifts corresponded to lower yellow intensity, possibly due to carotenoid degradation. The lack of significant correlation between b and antioxidant capacity (DPPH, ABTS) suggests that yellow pigments contribute minimally to antioxidant activity. Ozay-Arancioglu et al. (2021) investigated the drying process of pomegranate seeds and found that freeze-drying effectively minimized carotenoid degradation while maintaining superior color stability. In contrast, hot-air drying and microwave drying resulted in more pronounced losses of yellow pigments [63]. The a/b ratio, positively correlated with L* and negatively correlated with b*, reflects a shift from yellow to red while increasing overall brightness.** This metric may serve as an indicator of pigment composition changes, particularly the balance between polyphenol oxidation and Maillard reaction byproducts.

#### 3.9.2. TPC, TFC, and Key Bioactive Compounds

The relationships between TPC, TFC, and key bioactive compounds were comprehensively established, demonstrating strong interconnections. A highly significant positive correlation was observed between TPC and TFC (r = 0.97, *p* < 0.001), indicating that flavonoid-rich samples also possess higher levels of total phenolic compounds, thereby enhancing overall bioactivity. Moreover, chlorogenic acid, quercitrin, and eleutheroside E exhibited robust correlations with both TPC and TFC (r > 0.90, *p ≤* 0.05), suggesting their crucial role in the retention and accumulation of bioactive compounds in AS fruit.

Protocatechuic acid and eleutheroside E displayed a strong correlation (r = 0.92, *p ≤* 0.05), implying that these compounds may share similar biosynthetic pathways during metabolic processes. Additionally, chlorogenic acid exhibited significant positive correlations with quercitrin and eleutheroside B (r = 0.99 and 0.94, *p ≤* 0.05), suggesting potential similarities in their accumulation and metabolic patterns. Furthermore, rutin and hyperoside were found to be perfectly correlated (r = 1.0, *p* < 0.001), further reinforcing the hypothesis of a closely linked biosynthetic or regulatory mechanism governing their accumulation. Studies on freeze-dried pomegranate peel and apple pomace have also revealed a strong interdependence between flavonoid components, highlighting the stability of their biosynthetic pathways during drying processes [64].

#### 3.9.3. Amino Acids

Amino acids also exhibit significant interdependencies, indicating potential metabolic and biosynthetic interactions. Asp demonstrated strong positive correlations with Ser (r = 0.98, *p* < 0.01), Val (r = 0.90, *p ≤* 0.05), Tyr (r = 0.90, *p ≤* 0.05), and Phe (r = 0.90, *p ≤* 0.05), suggesting a coordinated role in amino acid metabolism. Asp is a key precursor in the biosynthesis of Ser and Val. Meanwhile, its metabolic connections with the biosynthesis of Tyr and Phe may explain their strong positive correlations [65]. Similarly, Ser exhibited significant correlations with Val (r = 0.94, *p ≤* 0.05), Ile (r = 0.90, *p ≤* 0.05), Tyr (r = 0.96, *p* < 0.01), and Phe (r = 0.94, *p ≤* 0.05). Ser is synthesized from 3-phosphoglycerate (a glycolytic intermediate) and plays a crucial role in cellular metabolism [66]. Its metabolic interactions with the biosynthesis of Val, Ile, Tyr, and Phe may explain their significant co-regulation. Additionally, Glu and His exhibited a strong positive correlation (r = 0.95, *p ≤* 0.05). Glu and His share metabolic links, as Glu serves as an essential nitrogen donor in His biosynthesis [67]. Gly was positively correlated with Val (r = 0.92, *p ≤* 0.05) and Phe (r = 0.84, *p ≤* 0.05), further supporting the existence of metabolic interactions. Gly plays a central role in one-carbon metabolism and is involved in metabolic pathways that influence the biosynthesis of Val and Phe, potentially explaining their positive correlations [68]. Val and Ile showed a highly significant correlation (r = 0.96, *p* < 0.01), as did Val and Phe (r = 1.00, *p* < 0.001). Ile also exhibited significant positive correlations with Leu (r = 0.93, *p ≤* 0.05) and Phe (r = 0.98, *p* < 0.01). Ile and Leu are synthesized through overlapping enzymatic pathways, including acetolactate synthase and branched-chain amino acid aminotransferase. Notably, Lys exhibited a significant negative correlation with eleutheroside E (r = −0.91, *p ≤* 0.05) and TFC (r = −0.88, *p ≤* 0.05). The observed inverse relationship suggests competition for metabolic resources, including enzymatic activity and carbon flux allocation, between amino acid biosynthesis and secondary metabolite production. Specifically, Lys biosynthesis follows the aspartate-derived pathway, whereas flavonoids and phenolics originate from the phenylpropanoid pathway. Although these pathways do not share direct precursors, their regulation and metabolic flux distribution may underlie this inverse correlation. Under stress conditions, plants often prioritize secondary metabolite production to enhance adaptive responses. Consequently, Lys biosynthesis may be downregulated due to limited substrate availability or shifts in carbon–nitrogen metabolic balance [69].

#### 3.9.4. Antioxidant Activity

Regarding antioxidant activity, both DPPH and ABTS radical scavenging capacity exhibited strong positive correlations with TFC (r > 0.92, *p ≤* 0.05) and TPC (r > 0.98, p *≤* 0.05). These results confirm that phenolic compounds play a pivotal role in the antioxidant potential of AS fruit. Furthermore, chlorogenic acid and eleutherosides demonstrated highly significant positive correlations with DPPH (r > 0.97, *p* < 0.01) and ABTS (r > 0.92, *p ≤* 0.05), further reinforcing their role as key contributors to the antioxidant properties of AS fruit. These findings demonstrate that the retention of these bioactive compounds is crucial for maximizing the functional value of AS fruit.

## 4. Discussion

This study comprehensively evaluated the influence of drying methods on color, bioactive compound retention, antioxidant activity, and metabolomic profiles in AS fruits, highlighting VFD as optimal across multiple quality parameters.

Color, a vital indicator of quality, was best preserved by VFD, as reflected by superior L* values and reduced ΔE. The low-temperature, low-oxygen conditions of VFD effectively suppressed non-enzymatic browning and polyphenol oxidation. Similar advantages were reported by Wojdyło et al. (2020), who observed enhanced color retention in freeze-dried apples [70]. Conversely, HD significantly accelerated Maillard reactions and polyphenol oxidation, negatively affecting color.

VFD achieved the highest retention of TPC, TFC, and major phenolic and flavonoid compounds. Low-temperature drying processes mitigate the oxidation and degradation of phenolic compounds, whereas HD and MD significantly reduced these heat-sensitive components. Dadhaneeya et al. (2023) also noted significant losses of phenolics and fiber in dragon fruit following hot-air drying [71]. Although VD avoids high temperatures, extended drying times may cause partial compound degradation. These results underscore the importance of controlling temperature and drying environment to preserve active components effectively.

Amino acid analysis showed VFD notably preserved heat-sensitive amino acids such as Lys, Arg, and Glu. Lower temperatures reduce amino acid degradation kinetics, enhancing compound integrity. Lu et al. (2024) similarly reported that VFD maintained higher amino acid-related metabolites in mushrooms compared with HD [72]. Additionally, PCA and OPLS-DA clearly demonstrated that drying methods significantly altered metabolomic profiles. VFD-treated samples formed distinct metabolite clusters, indicative of effective preservation, whereas high-temperature treatments caused notable degradation or transformation of thermolabile metabolites.

Antioxidant activity, a crucial parameter for functional foods, was highest in VFD-treated samples, as reflected by elevated DPPH and ABTS scavenging capacities. This outcome correlates directly with VFD’s superior retention of polyphenolic and flavonoid constituents, thus limiting oxidative damage. Recent studies consistently support freeze-drying’s superior retention of phenolics, flavonoids, and antioxidant activity. Freeze-dried plant materials exhibited greater antioxidant potential compared to heat-based drying methods [73,74]. Conversely, HD and MD significantly compromised antioxidant activity due to heat-induced oxidative degradation, as corroborated by Wongklom 2018, who reported lower antioxidant capacities in oven- and sun-dried samples [75].

Despite systematically evaluating the impact of different drying methods on the quality parameters, bioactive constituents, and volatile profiles of AS fruits, this study has several inherent limitations that warrant further investigation.

There are some limitations in the chemical assays for phenolic and flavonoid quantification. Chemical assays commonly used for quantifying phenolics and flavonoids have inherent limitations. The Folin–Ciocalteu reagent is not specific to phenolics, interacting broadly with various reducing substances such as ascorbic acid, amino acids, and reducing sugars, potentially leading to the overestimation of total phenolic content (TPC) [76]. Nonetheless, it remains useful for the comparative, semi-quantitative evaluation of drying methods. Similarly, the aluminum chloride (AlCl_3_) method for TFC depends on flavonoid complexation with aluminum ions, but certain flavonoids lacking suitable hydroxyl groups may not react efficiently, causing false negatives [77]. Conversely, interference by organic acids and other polyphenols can yield false positives, inflating flavonoid content measurements [78]. The high correlations among TPC, TFC, and antioxidant activity measurements are anticipated, as these assays share underlying redox chemistry. Specifically, DPPH and ABTS assays primarily evaluate radical scavenging, inherently linked to polyphenolic content. Consequently, drying techniques affecting polyphenol stability naturally impact antioxidant capacities. Future studies should employ complementary analytical techniques, such as HPLC or UPLC, enabling precise identification and quantification of individual bioactive phenolic and flavonoid compounds, thus minimizing assay-related interference.

The characterization of volatile profiles in this study was primarily conducted using PCA, which accounted for 64.2% of the total variance (PC1: 35.9%, PC2: 28.3%). Although PCA effectively captured major compositional trends, it did not reach the typically recommended threshold (≥70%) for robust dimensionality reduction, likely due to the inherent complexity and diverse responses of volatile compounds to drying treatments. Moreover, variance distribution and interpretability may be affected by data normalization and scaling methods. Alternative statistical techniques such as OPLS-DA or t-distributed Stochastic Neighbor Embedding (t-SNE) could provide superior class separation and deeper insights into the volatile changes induced by drying. A previous study found that PCA explained 66.47% variance in strawberry tree fruit volatile analysis, emphasizing the importance of complementary statistical approaches for interpreting complex metabolomic data [79]. Future studies should incorporate these methods to enhance the robustness and accuracy of volatile profiling.

Constraints of fixed drying parameters. This study evaluated five drying methods under fixed experimental conditions; however, bioactive compound retention and drying efficiency depend on multiple parameters, including temperature gradients, drying duration, airflow, and humidity. The standardized conditions applied here might not fully capture these dynamic interactions, potentially limiting insights into the retention mechanisms of phenolics, flavonoids, and volatile compounds. Recent studies emphasize the importance of optimizing drying parameters using advanced statistical and computational methods. A recent study optimized the hybrid microwave–hot-air drying of sweet potatoes using response surface methodology (RSM), demonstrating that optimal conditions can significantly improve drying time and rehydration ability and minimize bioactive compound loss [80]. Furthermore, machine learning approaches have been increasingly employed to enhance drying efficiency. Artificial neural networks (ANNs) combined with genetic algorithms (GAs) were applied to optimize the spray drying of probiotic beverages, demonstrating their effectiveness in modeling and optimizing drying parameters to enhance bioactive compound retention [81]. Future studies should, thus, explore broader drying parameter ranges using optimization techniques such as RSM or machine learning to determine conditions that best preserve bioactive compounds in AS fruits. Integrating these advanced approaches will facilitate the development of more efficient drying processes tailored for nutraceutical applications.

Lack of long-term stability assessments. This study primarily addressed the immediate impact of drying methods on AS fruit quality, neglecting the critical factor of post-processing stability. Bioactive compounds can degrade significantly over time due to oxidative, enzymatic, and structural changes, potentially affecting long-term efficacy and bioavailability. Storage conditions were found to have a significant impact on the stability of anthocyanin and ascorbic acid in dried raspberry products [82]. Future studies should, therefore, include accelerated aging tests and long-term storage trials to better understand phenolic, flavonoid, antioxidant, and volatile compound stability, ensuring improved shelf-life predictions and functional quality of dried AS fruits.

Generalizability and sample representativeness. A key limitation of this study is the use of AS fruits collected from a single geographic location (Northeast Forestry University, China) at one maturity stage, restricting the generalizability of the findings. Plant provenance, genetic variability, and environmental factors significantly affect bioactive compound accumulation and post-harvest responses to drying treatments. Previous studies confirmed that temperature fluctuations, photoperiod, soil composition, and environmental stressors (e.g., drought, UV radiation, salinity) substantially modulate secondary metabolite profiles in plants [83,84]. Additionally, regional variability markedly influences phenolic content and antioxidant capacity [85,86]. Future studies should incorporate multi-location, multi-season, and multi-maturity sampling, as well as different cultivars, to enhance the robustness, representativeness, and applicability of the conclusions across diverse environmental conditions.

Limited scope of biological activity assessments. This study assessed primary bioactive indicators (TPC, TFC, and antioxidant activity via DPPH and ABTS assays) but did not explore other significant biological activities relevant to functional applications of AS fruits. Recent studies indicate that AS fruits may possess additional bioactivities, including anti-inflammatory, antimicrobial, hypoglycemic, immunomodulatory, cardiovascular-protective, and hepatoprotective effects [87,88,89]. Future research should, therefore, include comprehensive in vitro and in vivo evaluations using cellular and animal models to thoroughly characterize the bioactivities influenced by different drying methods.

## 5. Conclusions

This study demonstrated differences in AS fruit quality depending on drying methods, with VFD consistently superior in preserving bioactive components and overall quality. VFD-treated fruits maintained the highest total phenolic and flavonoid contents (e.g., chlorogenic acid, rutin, quercitrin), essential amino acids, and antioxidant activities. Furthermore, VFD effectively minimized color degradation, closely matching fresh fruit characteristics.

ND preserved bioactive compounds moderately well due to low-temperature exposure, resulting in antioxidant activity second only to VFD; however, prolonged drying time and susceptibility to contamination limit its industrial applicability. MD provided intermediate results, retaining bioactive compounds and antioxidant activities better than HD but inferior to VFD, reflecting moderate thermal damage.

Conversely, HD led to considerable degradation of phenolic compounds, volatiles, and severe color deterioration, significantly reducing antioxidant capacity. VD showed the poorest retention of bioactive constituents and antioxidant activity (approximately one-fifth to one-sixth of VFD), marking it as the least suitable drying technique for AS fruits.

Overall, drying methods ranked as follows in preserving AS fruit quality: VFD (optimal) > ND, MD, HD (moderate) > VD (least recommended).

## Figures and Tables

**Table 1:** Effects of different drying methods on the color parameters of AS fruits.

Sample Code	HD	VD	ND	MD	VFD	Fresh Fruit
L*	22.66 ± 0.56 ^f^	30.36 ± 0.26 ^c^	26.92 ± 0.18 ^e^	27.41 ± 0.22 ^d^	33.56 ± 0.23 ^a^	32.01 ± 0.16 ^b^
a*	4.91 ± 0.10 ^a^	1.25 ± 0.10 ^d^	3.17 ± 0.15 ^b^	2.18 ± 0.14 ^c^	0.52 ± 0.02 ^e^	−1.12 ± 0.04 ^f^
b*	−7.11 ± 0.03 ^d^	−4.37 ± 0.19 ^c^	−6.42 ± 0.08 ^d^	−6.38 ± 0.05 ^d^	−3.33 ± 0.21 ^b^	−2.45 ± 0.11 ^a^
a*/b*	−0.69 ± 0.01 ^f^	−0.29 ± 0.03 ^c^	−0.49 ± 0.02 ^e^	−0.34 ± 0.02 ^b^	−0.16 ± 0.01 ^d^	0.46 ± 0.02 ^a^
ΔE	11.11 ± 0.50 ^a^	2.53 ± 0.20 ^d^	6.77 ± 0.26 ^b^	6.14 ± 0.24 ^c^	1.88 ± 0.33 ^e^	0.00
MC%	9.48 ± 0.02 ^c^	8.54 ± 0.12 ^d^	14.97 ± 0.12 ^b^	6.63 ± 0.18 ^e^	5.91 ± 0.14 ^f^	82.95 ± 0.28 ^a^

Values are expressed as mean ± standard deviation. Different small letters in the same column indicate significant differences (*p ≤* 0.05).

**Table 2:** Analysis of variance in active ingredients of AS fruits subjected to different drying methods by UHPLC-UV.

Sample Code	HD	VD	ND	MD	VFD
Protocatechuic acid (mg/100 g)	5.4 ± 0.3 ^a^	1.8 ± 0.2 ^d^	3.5 ± 0.1 ^b^	3.6 ± 0.1 ^b^	2.5 ± 0.2 ^c^
Chlorogenic acid (mg/100 g)	294 ± 2 ^d^	71.6 ± 0.2 ^e^	499 ± 1 ^b^	486 ± 8 ^c^	601 ± 6 ^a^
Rutin (mg/100 g)	15.5 ± 0.3 ^b^	6.7 ± 0.6 ^d^	6.9 ± 0.3 ^d^	13.9 ± 0.4 ^c^	23.3 ± 0.6 ^a^
Hyperoside (mg/100 g)	72.0 ± 1.5 ^b^	40.0 ± 0.4 ^e^	42.3 ± 1.6 ^d^	68.1 ± 3.7 ^c^	96.6 ± 1.2 ^a^
Isofraxidin (mg/100 g)	2.32 ± 0.03 ^a^	0.52 ± 0.03 ^d^	1.28 ± 0.03 ^c^	0.49 ± 0.02 ^d^	1.67 ± 0.05 ^b^
Quercitrin (mg/100 g)	238 ± 5 ^d^	66.9 ± 1.3 ^e^	434 ± 4 ^b^	351 ± 5 ^c^	535 ± 6 ^a^
Eleutheroside B (mg/100 g)	65.9 ± 1.1 ^c^	21.0 ± 0.3 ^d^	124 ± 1 ^b^	69.2 ± 3.8 ^c^	162 ± 2 ^a^
Eleutheroside E (mg/100 g)	20.5 ± 0.5 ^a^	11.8 ± 0.2 ^d^	16.4 ± 0.4 ^c^	17.3 ± 0.7 ^b^	16.4 ± 0.2 ^c^

Different letters (a–e) indicate significant differences (*p ≤* 0.05) among drying methods within the same row (same chemical compound).

**Table 3:** Analysis of variance in amino acid composition of AS fruits subjected to different drying methods.

Amino Acid (mg/g)	HD	VD	ND	MD	VFD
Asp	0.54 ± 0.02 ^b^	0.48 ± 0.01 ^c^	0.14 ± 0.01 ^e^	0.31 ± 0.01 ^d^	0.57 ± 0.02 ^a^
Thr	0.95 ± 0.03 ^c^	1.83 ± 0.06 ^b^	0.72 ± 0.02 ^d^	0.92 ± 0.03 ^cd^	3.54 ± 0.11 ^a^
Ser	0.62 ± 0.02 ^b^	0.55 ± 0.02 ^c^	0.17 ± 0.01 ^e^	0.26 ± 0.01 ^d^	0.71 ± 0.02 ^a^
Glu	0.58 ± 0.02 ^b^	0.31 ± 0.01 ^ce^	0.49 ± 0.02 ^c^	0.37 ± 0.01 ^d^	0.90 ± 0.03 ^a^
Gly	0.073 ± 0.006 ^ab^	0.080 ± 0.002 ^a^	0.024 ± 0.006 ^cd^	0.027 ± 0.011 ^cd^	0.050 ± 0.002 ^bc^
Ala	0.88 ± 0.03 ^a^	0.37 ± 0.01 ^b^	0.29 ± 0.01 ^bc^	0.37 ± 0.02 ^b^	0.28 ± 0.01 ^bc^
Cys	0.082 ± 0.011 ^c^	0.090 ± 0.003 ^bc^	0.094 ± 0.005 ^b^	0.088 ± 0.007 ^b c^	0.110 ± 0.003 ^a^
Val	0.34 ± 0.01 ^b^	0.36 ± 0.01 ^a^	0.13 ± 0.02 ^c^	0.12 ± 0.01 ^c^	0.34 ± 0.01 ^b^
Met	n.d.	n.d.	n.d.	n.d.	0.040 ± 0.001 ^a^
Ile	0.19 ± 0.01 ^c^	0.28 ± 0.01 ^a^	0.064 ± 0.005 ^d^	0.060 ± 0.002 ^d^	0.26 ± 0.01 ^b^
Leu	0.18 ± 0.01 ^c^	0.45 ± 0.01 ^a^	0.074 ± 0.012 ^e^	0.090 ± 0.003 ^d^	0.29 ± 0.01 ^b^
Tyr	0.18 ± 0.01 ^b^	0.17 ± 0.01 ^bc^	0.13 ± 0.01 ^cd^	0.13 ± 0.01 ^cd^	0.22 ± 0.01 ^a^
Phe	0.31 ± 0.01 ^c^	0.38 ± 0.01 ^a^	0.023 ± 0.006 ^d^	0.030 ± 0.001 ^d^	0.34 ± 0.01 ^b^
Lys	0.026 ± 0.006 ^cd^	0.21 ± 0.01 ^a^	0.054 ± 0.005 ^bc^	0.010 ± 0.000 ^de^	0.060 ± 0.002 ^b^
His	0.090 ± 0.003 ^b^	0.060 ± 0.002 ^d^	0.074 ± 0.005 ^c^	0.030 ± 0.001 ^e^	0.180 ± 0.005 ^a^
Arg	2.63 ± 0.08 ^c^	2.07 ± 0.08 ^d^	2.90 ± 0.09 ^b^	1.07 ± 0.03 ^e^	3.61 ± 0.11 ^a^
Pro	0.29 ± 0.01 ^a^	0.16 ± 0.01 ^c^	0.25 ± 0.01 ^b^	0.050 ± 0.002 ^e^	0.090 ± 0.003 ^d^

Results are expressed as mean ± standard error (SE). Mean values within the same column followed by different letters (a–e) indicate statistically significant differences (*p ≤* 0.05) among drying methods. “n.d.” denotes not detected.

## Data Availability

The original contributions presented in this study are included in the article/Supplementary Materials. Further inquiries can be directed to the corresponding author.

## Supplementary Materials

The following supporting information can be downloaded at: https://www.mdpi.com/article/10.3390/foods14071100/s1, Table S1. Separation gradient conditions for liquid chromatography. Table S2. Total Phenolic Content (TPC) and Total Flavonoid Content (TFC) in AS fruits subjected to different drying methods. Table S3. Calibration Curve Parameters and Recovery Rates of Components. Table S4. Identification and Characterization of Volatile Compounds Based on Mass Spectrometry and Retention Indices in AS Fruits Treated with Different Drying Methods. Table S5. Analysis of volatile compounds with VIP > 1.0 in AS fruits treated with different drying methods. Table S6. The compounds that meet the dual criteria of VIP > 1 and *p* < 0.05 in AS fruits treated with different drying methods. Table S7. Analysis of Antioxidant Activity in AS fruits treated with different drying methods. Figure S1. Chromatogram of the standards solution. The chromatographic peaks were numbered as follows: (1) protocatechuic acid, (2) eleutheroside B, (3) chlorogenic acid, (4) eleutheroside E, (5) quercitrin, (6) hyperoside, (7) isofraxidin, and (8) rutin. Figure S2. Chromatogram of the VFD sample. The chromatographic peaks were numbered as follows: (1) protocatechuic acid, (2) eleutheroside B, (3) chlorogenic acid, (4) eleutheroside E, (5) quercitrin, (6) hyperoside, (7) isofraxidin, and (8) rutin. Figure S3. Principal components analysis of main active ingredients of AS fruit under five different drying methods. Figure S4. Partial Least Squares Discriminant Analysis of volatile compounds in AS fruit subjected to five different drying methods. Figure S5. Variable importance in projection (VIP) values of characteristic volatile compounds (a) and permutation test results (b) for volatile compounds in five groups of AS fruit samples subjected to different drying methods.
