# Supplementary material for: Evaluation of Different Drying Methods on the Quality Parameters of Acanthopanax senticosus Fruits
Source: Foods. 2025 Mar 22;14(7):1100. doi: 10.3390/foods14071100 (PMC11989058; doi:10.3390/foods14071100)
Supplement: Supplementary file 1 [file foods-14-01100-s001.zip › foods-3515645-supplementary.pdf]

Supplementary Materials:

Table S1. Separation gradient conditions for liquid chromatography.

No	Time (min)	Flow rate (mL/min)	A (%)	B (%)
1	0	0.300	95.0	5.0
2	3	0.300	85.5	14.5
3	4	0.300	83.5	16.5
4	7	0.300	57.0	43.0
5	7.5	0.300	0.0	100.0
6	12	0.300	0.0	100.0
7	13	0.300	95.0	5.0
8	15	0.300	95.0	5.0

Mobile phase A consisted of 0.4% (v/v) aqueous phosphoric acid, while mobile phase B was acetonitrile.

Table S2. Total Phenolic Content (TPC) and Total Flavonoid Content (TFC) in AS fruits subjected to different drying methods.

Group	HD	VD	ND	MD	VFD
TFC (mg/g)	78.3 ± 5.4[c]	11.4 ± 1.0[d]	122.2 ± 4.0[a]	90.0 ± 5.0[b]	131.9 ± 3.5[a]
TPC (mg/g)	8.93 ± 1.79[c]	0.93 ± 0.11[d]	19.60 ± 1.22[b]	15.26 ± 3.36[b]	23.62 ± 1.57[a]

Values are expressed as mean ± standard deviation (SD). Different lowercase letters within the same column indicate significant differences at $p < 0.05$.

Table S3. Calibration Curve Parameters and Recovery Rates of Components

Component(s)	Calibration curve equation	Linear range (μg/ml)	R^2	Detection limits (ng/ml)	High recovery (%)	Medium recovery (%)	Low recovery (%)
Protocatechuic acid	Y = 15049X +7132.4	16.20-81.00	0.9999	21.93	98.2 ± 1.1	97.0 ± 1.3	96.5 ± 1.5
Chlorogenic acid	Y = 5216.1X +4823.2	11.96-59.80	0.9998	63.27	99.5 ± 0.9	98.6 ± 1.2	97.4 ± 1.6
Rutin	Y = 16285X +9743.7	12.46-62.30	0.9999	20.26	100.3 ± 0.8	99.2 ± 1.0	98.1 ± 1.4
Hyperoside	Y = 7557.4X +5804.6	11.16-55.80	1	43.67	101.0 ± 1.2	98.5 ± 1.4	99.3 ± 1.8
Isofraxidin	Y = 21118X +13715	9.62-48.10	1	15.63	98.7 ± 1.0	97.8 ± 1.2	97.0 ± 1.5
Quercitrin	Y = 3682.1X +4758.8	21.24-107.00	1	89.62	97.5 ± 1.3	96.8 ± 1.5	95.6 ± 1.9

Eleutheroside B	Y = 24478X +16058	7.78-39.00	0.9998	13.48	99.8 ± 0.7	99.0 ± 1.1	98.0 ± 1.3
Eleutheroside E	Y = 6721.1X +7603.1	10.80-54.00	0.9965	49.10	96.6 ± 0.9	99.7 ± 1.3	98.6 ± 1.5

The limit of detection (LOD) was calculated using the regression equation method, as recommended by the ICH guidelines: LOD = 3.3 × σ / s, where σ represents the standard deviation of baseline noise (mAU) and s denotes the slope of the standard curve (mAU/concentration).

foods

MDPI

Table S4. Identification and Characterization of Volatile Compounds Based on Mass Spectrometry and Retention Indices in AS Fruits Treated with Different Drying Methods

No	Volatile compound	Molecular	Cas	Molecular Weight	M/z ratios	Match scores (HD,VD,ND,MD,VFD)	Retention Index
1	.alpha.-Phellandrene	C10H16	99-83-2	136.23	136[M]+,93,92,91,77	93.24,93.22,90.44,93.28,93.40	1005
2	Cyclohexene, 1-methyl-4-(1-methylethylidene)-	C10H16	586-62-9	136.23	94[M]+,93,91	93.28,93.80,89.76,93.56,92.23	1080
3	(1R)-2,6,6-Trimethylbicyclo[3.1.1]hept-2-ene	C10H16	7785-70-8	136.23	94[M]+,9.,92,79,43	89.15,91.37,88.26,92.84,93.15	939
4	3-Carene	C10H16	13466-78-9	136.23	92[M]+,92,91,79,77	94.88,94.60,91.51,94.59,92.99	1004
5	Bicyclo[3.1.0]hex-2-ene, 2-methyl-5-(1-methylethyl)-	C10H16	2867-05-2	136.23	98[M]+,93,92,91,77	79.84,83.64,84.47,82.14,80.69	1376
6	.gamma.-Terpinene	C10H16	99-85-4	136.23	136[M]+,121,93,91,77	94.14,94.53,93.46,92.83,93.11	1054

7	1,3-Cyclohexadiene, 1-methyl-4-(1-methylethyl)-	C10H16	99-86-5	136.23	136[M]+,121,93,91,79,77	88.34,85.49,85.16,87.57,87.76	1020
8	2,4,6-Octatriene, 2,6-dimethyl-, (E,Z)-	C10H16	7216-56-0	136.23	136[M]+,121,105,93,91,79,77,41,39	89.97,87.48,86.66,85.37,88.55	1000
9	Cyclohexene, 4-ethenyl-4-methyl-3-(1-methylethenyl)-1-(1-methylethyl)-, (3R-trans)-	C15H24	20307-84-0	204.35	161[M]+,136,121,91,41	93.75,93.99,93.64,92.75,94.42	1450
10	3a,7-Methano-3aH-cyclopentacyclooctene, 1,4,5,6,7,8,9,9a-octahydro-1,1,7-trimethyl-, [3aR-(3a.alpha.,7.alpha.,9a.beta.)]-	C15H24	469-92-1	204.35	204[M]+,189,161,133,119,105	93.78,92.92,90.78,92.47,93.64	1376
11	.alfa.-Copaene	C15H24	3856-25-5	204.35	119[M]+,105,93,91,41	95.15,94.86,94.35,96.15,94.85	1054
12	(1R,3aS,8aS)-7-Isopropyl-1,4-dimethyl-1,2,3,3a,6,8a-hexahydroazulene	C15H24	36577-33-0	204.35	161[M]+,123,119,105	96.18,95.18,95.92,95.53,94.77	1012
13	Longifolene-(V4)	C15H24	61262-67-7	204.35	203[M]+,190,161,147,120,83	93.86,93.60,94.75,94.04,95.37	1410
14	(1R,2S,6S,7S,8S)-8-Isopropyl-1-methyl-3-methylenetricyclo[4.4.0.02,7]decane-rel-	C15H24	18252-44-3	204.35	204[M]+,189,145,53,43,41	94.41,94.55,93.30,95.53,93.92	1376
15	Aromandendrene	C15H24	489-39-4	204.35	204[M]+,189,161,147,133,119,107,105	93.36,93.09,94.29,91.57,92.85	1431
16	.gamma.-Muurolene	C15H24	24268-39-1	204.35	204[M]+,189,161,119,105,93,91	94.17,93.07,92.14,93.99,94.01	1012

17	Isocaryophillene	C15H24	1000140-07-2	204.35	133[M]+,100,93,79,	93.30,92.49,92.34,92.72, 92.88	1054
18	(+)-epi-Bicyclosesquiphellandrene	C15H24	54274-73-6	204.35	161[M]+,105,93,77	94.19,94.16,94.08,94.57, 94.76	1440
19	Aromadendrene, dehydro-	C15H22	1000156-12-5	202.34	202[M]+,159,145,107	82.70,82.80,83.41,82.32, 83.00	1580
20	Naphthalene, 1,2,3,5,6,8a-hexahydro-4,7-dimethyl-1-(1-methylethyl)-, (1S-cis)-	C15H24	483-76-1	204.35	161[M]+,119,105,100,91	92.31,92.12,87.91,93.02, 92.53	1420
21	1H-Cyclopropa[a]naphthalene, 1a,2,3,3a,4,5,6,7b-octahydro-1,1,3a,7-tetramethyl-, [1aR-(1a.alpha.,3a.alpha.,7b.alpha.)]-	C15H24	489-29-2	204.35	204[M]+,189，161,105	94.52,93.66,94.70,95.01, 92.21	1430
22	1H-Benzocycloheptene, 2,4a,5,6,7,8,9,9a-octahydro-3,5,5-trimethyl-9-methylene-, (4aS-cis)-	C15H24	3853-83-6	204.35	204[M]+,189，161,121,119	92.17,93.42,83.97,93.72, 92.43	1430
23	1H-3a,7-Methanoazulene, 2,3,4,7,8,8a-hexahydro-3,6,8,8-tetramethyl-, [3R-(3.alpha.,3a.beta.,7.beta.,8a.alpha.)]-	C15H24	469-61-4	204.35	204[M]+,189,161,119,105, 93	92.04,90.15,92.71,87.51, 91.78	1611
24	Naphthalene, 1,2,3,4,4a,5,6,8a-octahydro-7-methyl-4-methylene-1-(1-methylethyl)-, (1.alpha.,4a.beta.,8a.alpha.)-	C15H24	39029-41-9	204.35	204[M]+,189,161,135,119, 105	96.40,96.70,95.97,96.34, 96.33	1513
25	Naphthalene, 1,2,4a,5,8,8a-hexahydro-4,7-dimethyl-1-(1-methylethyl)-, [1S-(1.alpha.,4a.beta.,8a.alpha.)]-	C15H24	523-47-7	204.35	162[M]+,161,160,159	90.25,89.33,90.84,89.27, 88.54	1450
26	(Z)-1-Methyl-4-(6-methylhept-5-en-2-ylidene)cyclohex-1-ene	C15H24	13062-00-5	204.35	119[M]+,107.93.88	92.60,92.45,92.14,92.78, 92.37	1450

27	.alpha.-Muurolene	C15H24	10208-80-7	204.35	202[M]+,157,142,159	90.82,90.06,91.05,92.06, 91.25	1490
28	Cadala-1(10),3,8-triene	C15H22	1000140-05-6	202.34	202[M]+,157,142,200,159	75.11,74.49,75.09,73.32, 74.57	1580
29	Camphene	C10H16	79-92-5	136.23	137[M]+,121,136,105	91.16,90.16,92.35,92.23, 91.79	943
30	2,4,6-Octatriene, 3,4-dimethyl-	C10H16	57396-75-5	136.23	137[M]+,121,136,105,79	90.64,91.51,90.55,91.17, 92.05	
31	Tricyclo[5.4.0.0(2,8)]undec-9-ene, 2,6,6,9-tetramethyl-, (1R,2S,7R,8R)-	C15H24	5989-08-2	204.35	204[M]+119,105,138,93	92.21,93.03,92.16,93.36, 92.22	939
32	1H-Cycloprop[e]azulene, 1a,2,3,4,4a,5,6,7b-octahydro-1,1,4,7-tetramethyl-, [1aR-(1a.alpha.,4.alpha.,4a.beta.,7b.alpha.)]-	C15H24	489-40-7	204.35	204[M]+,189,161,119,105, 91,41	94.37,94.43,92.55,93.38, 94.33	1485
33	.alpha.-Farnesene	C15H24	502-61-4	204.35	203[M]+,188,69,67,55	87.99,87.86,85.24,84.41, 89.10	1506
34	.alpha.-Calacorene	C15H20	21391-99-1	200.92	158[M]+,157,156,155,154	81.10,80.90,82.34,82.90, 78.63	1620
35	3,5,11-Eudesmatriene	C15H22	193615-07-5	202.35	202[M]+187,161	87.09,88.01,88.57,87.39, 87.08	1580
36	.gamma.-HIMACHALENE	C15H24	1000140-08-0	204.35	137[M]+,133,105,93	89.36,89.20,90.59,89.78, 89.49	1480

37	1,3,5-Cycloheptatriene, 3,7,7-trimethyl-	C10H14	3479-89-8	134.22	134[M]+,119,91,62	93.54,93.36,92.02,91.55, 93.94	953
38	D-Limonene	C10H16	5989-27-5	136.23	68.1[M]+,15.22	95.66,93.90,94.73,92.45, 94.87	1018
39	1S,2S,5R-1,4,4-Trimethyltricyclo[6.3.1.0(2,5)]dodec-8(9)-ene	C15H24	1000140-07-5	204.35	204[M]+,101,93,41	93.47,93.33,93.51,94.93, 92.27	1450
40	Caryophyllene	C15H24	87-44-5	204.35	204[M]+,133,93,91,79,69	95.57,94.42,94.73,93.66, 94.67	1420
41	(1S,2E,6E,10R)-3,7,11,11-Tetramethylbicyclo[8.1.0]undeca-2,6-diene	C15H24	24703-35-3	204.35	121[M]+,93,70,41	94.27,93.54,94.22,93.38, 94.87	1570
42	1,4,7,-Cycloundecatriene, 1,5,9,9-tetramethyl-, Z,Z,Z-	C15H24	1000062-61-9	204.35	204[M]+,189,161,133	93.46,92.77,93.79,94.09, 93.62	1180
43	.beta.-Longipinene	C15H24	41432-70-6	204.35	105[M]+,93,91	87.64,87.39,88.51,87.26, 86.15	1600
44	Cyclohexane, 1-methyl-3-(1-methylethenyl)-, cis-	C10H18	24399-15-3	204.35	95[M]+,82.81.49	78.38,78.61,76.95,78.55, 77.91	1550
45	Bicyclo[5.3.0]decane, 2-methylene-5-(1-methylvinyl)-8-methyl-	C15H24	1000159-39-3	204.35	204[M]+,189,161,147,133, 107,93,79	94.37,93.68,94.88,94.41, 92.65	1485
46	4,4-Dimethyl-3-(3-methylbut-3-enylidene)-2-methylenebicyclo[4.1.0]heptane	C15H22	79718-83-5	204.35	145[M]+,139,131,105	89.70,88.63,85.72,85.67, 88.39	1510
47	Sabinene	C10H16	3387-41-5	136.23	100[M]+,93,91,77,63.4	93.62,93.33,92.50,93.34, 94.06	1030

48	Cyclohexane, 1-methylene-4-(1-methylethenyl)-	C10H16	499-97-8	136.23	94[M]+,93,91	92.86,92.46,88.42,91.13, 92.51	1670
49	Terpinen-4-ol	C10H18O	562-74-3	154.25	111.0[M]+,99.99,93.0	90.59,88.61,92.30,91.01, 91.97	1250
50	Bicyclo[3.1.0]hexan-3-ol, 4-methylene-1-(1-methylethyl)-, (1.alpha.,3.alpha.,5.alpha.)-	C10H16O	3310-02-9	152.23	92[M]+,91,77,43	91.62,91.82,90.37,92.04, 91.59	1480
51	Isospathulenol	C15H24O	88395-46-4	220.35	220[M]+,202,159,119,43,41	91.88,92.22,94.75,89.23, 92.56	1470
52	.alpha.-Bisabolol	C15H26O	515-69-5	222.37	204[M]+,161,119,109,69,43	92.92,93.38,92.66,93.53, 93.38	1700
53	Pulegone	C10H16O	89-82-7	152.23	152.1[M]+,109.1,81.1,67.1,41.1	72.83,73.05,72.41,72.99, 73.46	1470
54	1,13-Tetradecadien-3-one	C14H24O	58879-40-6	210.36	194[M]+,96,82,55,41	91.23,90.66,91.67,92.37, 90.00	1760
55	3-(O-Anisidinomethyl)-5-(3-fluorobenzylidene)-2,4-thiazolidinedione	C18H15FN2O3S	302954-96-7	358.39	119[M]+,117,74,46,45	85.78,87.64,85.04,86.65, 87.05	1110
56	2-Cyclohexen-1-one, 2-methyl-5-(1-methylethyl)-, (S)-	C10H16O	499-71-8	152.23	119[M]+,74,46,45,44	82.67,82.30,84.59,83.34, 82.08	920
57	Cis-bicyclo[4.4.0]decan-1-ol-3-one	C10H16O2	42393-93-1	168.23	111[M]+,98,65,55	79.96,79.00,80.09,78.57, 79.95	1054
58	(6,6-Dimethylbicyclo[3.1.1]hept-2-en-2-yl)methyl ethyl carbonate	C13H20O3	1000373-80-4	224.30	106[M]+,105,104	92.75,90.54,92.98,93.05, 92.45	1220

59	2-Octen-1-ol, 3,7-dimethyl-, isobutyrate, (Z)-	C13H20O3	1000132-45-6	224.30	106[M]+,105,104	75.54,75.94,74.54,76.67,<br>76.04	1460
60	Benzaldehyde	C7H6O	100-52-7	106.12	107[M]+,106,105,77,51,50	86.66,86.31,85.52,86.03,<br>86.39	1040

Table S5. Analysis of volatile compounds with VIP > 1.0 in AS fruits treated with different drying methods.

NO	Var ID (Primary)	VIP Score
46	4,4-Dimethyl-3-(3-methylbut-3-enylidene)-2-methylenebicyclo[4.1.0]heptane	1.18174
35	3,5,11-Eudesmatriene	1.15041
15	Aromandendrene	1.14422
40	Caryophyllene	1.11414
10	3a,7-Methano-3aH-cyclopentacyclooctene, 1,4,5,6,7,8,9,9a-octahydro-1,1,7-trimethyl-, [3aR-(3a.alpha.,7.alpha.,9a.beta.)]-	1.10971
43	.beta.-Longipinene	1.09495
23	1H-3a,7-Methanoazulene, 2,3,4,7,8,8a-hexahydro-3,6,8,8-tetramethyl-, [3R-(3.alpha.,3a.beta.,7.beta.,8a.alpha.)]-	1.07762
51	Isospathulenol	1.06991
49	Terpinen-4-ol	1.06507
42	1,4,7,-Cycloundecatriene, 1,5,9,9-tetramethyl-, Z,Z,Z-	1.0631
28	Cadala-1(10),3,8-triene	1.06074
37	1,3,5-Cycloheptatriene, 3,7,7-trimethyl-	1.05688
39	1S,2S,5R-1,4,4-Trimethyltricyclo[6.3.1.0(2,5)]dodec-8(9)-ene	1.05688
4	3-Carene	1.05659
21	1H-Cyclopropa[a]naphthalene, 1a,2,3,3a,4,5,6,7b-octahydro-1,1,3a,7-tetramethyl-, [1aR-(1a.alpha.,3a.alpha.,7b.alpha.)]-	1.04285
45	Bicyclo[5.3.0]decane, 2-methylene-5-(1-methylvinyl)-8-methyl-	1.03325
20	Naphthalene, 1,2,3,5,6,8a-hexahydro-4,7-dimethyl-1-(1-methylethyl)-, (1S-cis)-	1.03081
54	1,13-Tetradecadien-3-one	1.02816
25	Naphthalene, 1,2,4a,5,8,8a-hexahydro-4,7-dimethyl-1-(1-methylethyl)-, [1S-(1.alpha.,4a.beta.,8a.alpha.)]-	1.0256
47	Sabinene	1.02299

29	Camphene	1.02109
18	(+)-epi-Bicyclosesquiphellandrene	1.02103
17	Isocaryophillene	1.01379
8	2,4,6-Octatriene, 2,6-dimethyl-, (E,Z)-	1.01345
44	Cyclohexane, 1-methyl-3-(1-methylethenyl)-, cis-	1.01031
27	.alpha.-Muurolene	1.00629
16	.gamma.-Muurolene	1.00509
9	Cyclohexene, 4-ethenyl-4-methyl-3-(1-methylethenyl)-1-(1-methylethyl)-, (3R-trans)-	1.00053

Table S6. The compounds that meet the dual criteria of VIP > 1 and $p < 0.05$ in AS fruits treated with different drying methods.

NO	Compound Name
10	3a,7-Methano-3aH-cyclopentacyclooctene, 1,4,5,6,7,8,9,9a-octahydro-1,1,7-trimethyl-, [3aR-(3a.alpha.,7.alpha.,9a.beta.)]-
15	Aromandendrene
17	Isocaryophillene
20	Naphthalene, 1,2,3,5,6,8a-hexahydro-4,7-dimethyl-1-(1-methylethyl)-, (1S-cis)-
23	1H-3a,7-Methanoazulene, 2,3,4,7,8,8a-hexahydro-3,6,8,8-tetramethyl-, [3R-(3.alpha.,3a.beta.,7.beta.,8a.alpha.)]-
25	Naphthalene, 1,2,4a,5,8,8a-hexahydro-4,7-dimethyl-1-(1-methylethyl)-, [1S-(1.alpha.,4a.beta.,8a.alpha.)]-
28	Cadala-1(10),3,8-triene
40	Caryophyllene
44	Cyclohexane, 1-methyl-3-(1-methylethenyl)-, cis-
46	4,4-Dimethyl-3-(3-methylbut-3-enylidene)-2-methylenebicyclo[4.1.0]heptane
51	Isospathulenol
54	1,13-Tetradecadien-3-one

Table S7. Analysis of Antioxidant Activity in AS fruits treated with different drying methods.

Group	HD	VD	ND	MD	VFD
DPPH	40.7 ± 0.7^{e}	11.5 ± 0.1^{d}	59.7 ± 1.5^{b}	46.8 ± 2.1^{c}	65.3 ± 2.6^{a}

ABTS	14.6 ± 0.3[d]	4.3 ± 0.1[e]	25.1 ± 1.1[b]	21.6 ± 0.7[c]	32.1 ± 0.6[a]

Values are expressed as mean ± standard deviation. Different small letters in the same column indicate significant differences ($p < 0.05$).

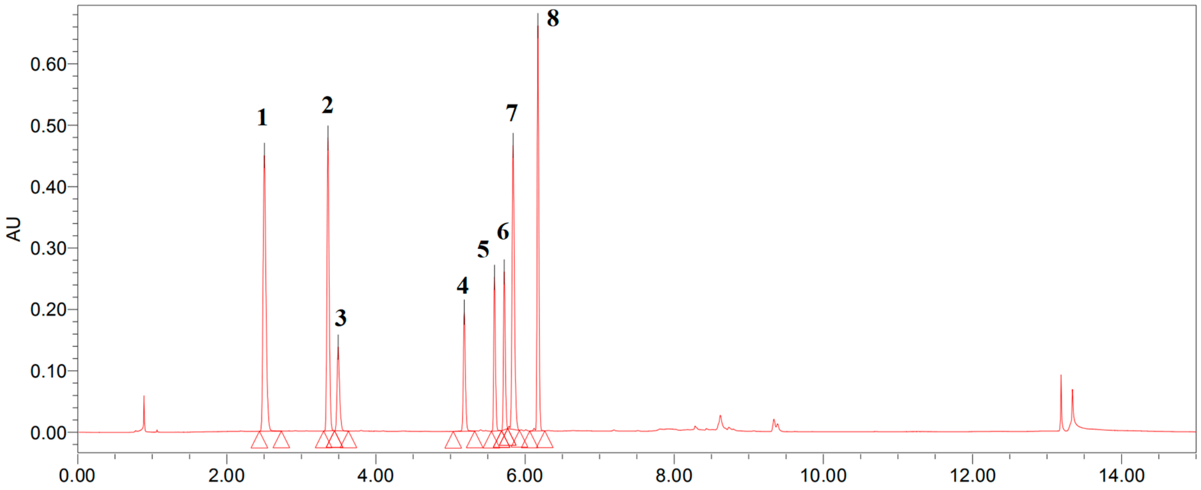

Figure S1. Chromatogram of the standards solution. The chromatographic peaks were numbered as follows: (1) protocatechuic acid, (2) eleutheroside B, (3) chlorogenic acid, (4) eleutheroside E, (5) quercitrin, (6) hyperoside, (7) isofraxidin, and (8) rutin.

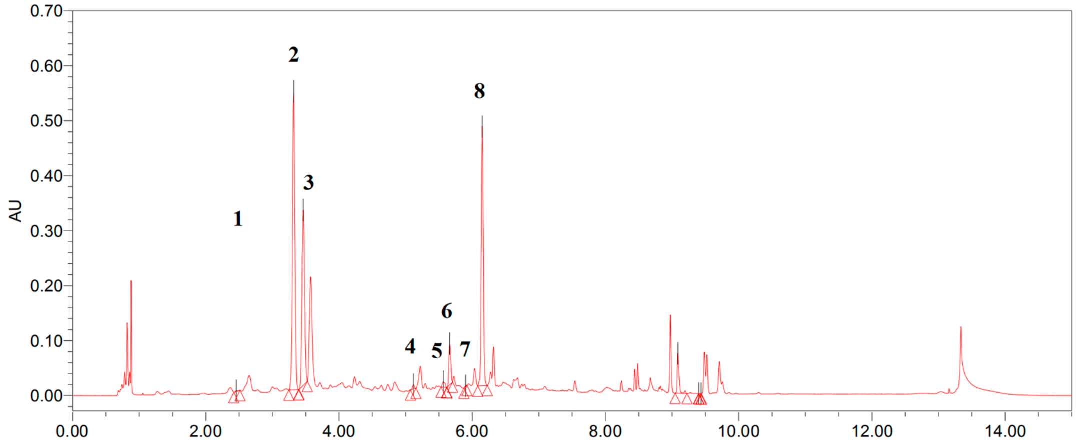

Figure S2. Chromatogram of the VFD sample. The chromatographic peaks were numbered as follows: (1) protocatechuic acid, (2) eleutheroside B, (3) chlorogenic acid, (4) eleutheroside E, (5) quercitrin, (6) hyperoside, (7) isofraxidin, and (8) rutin.

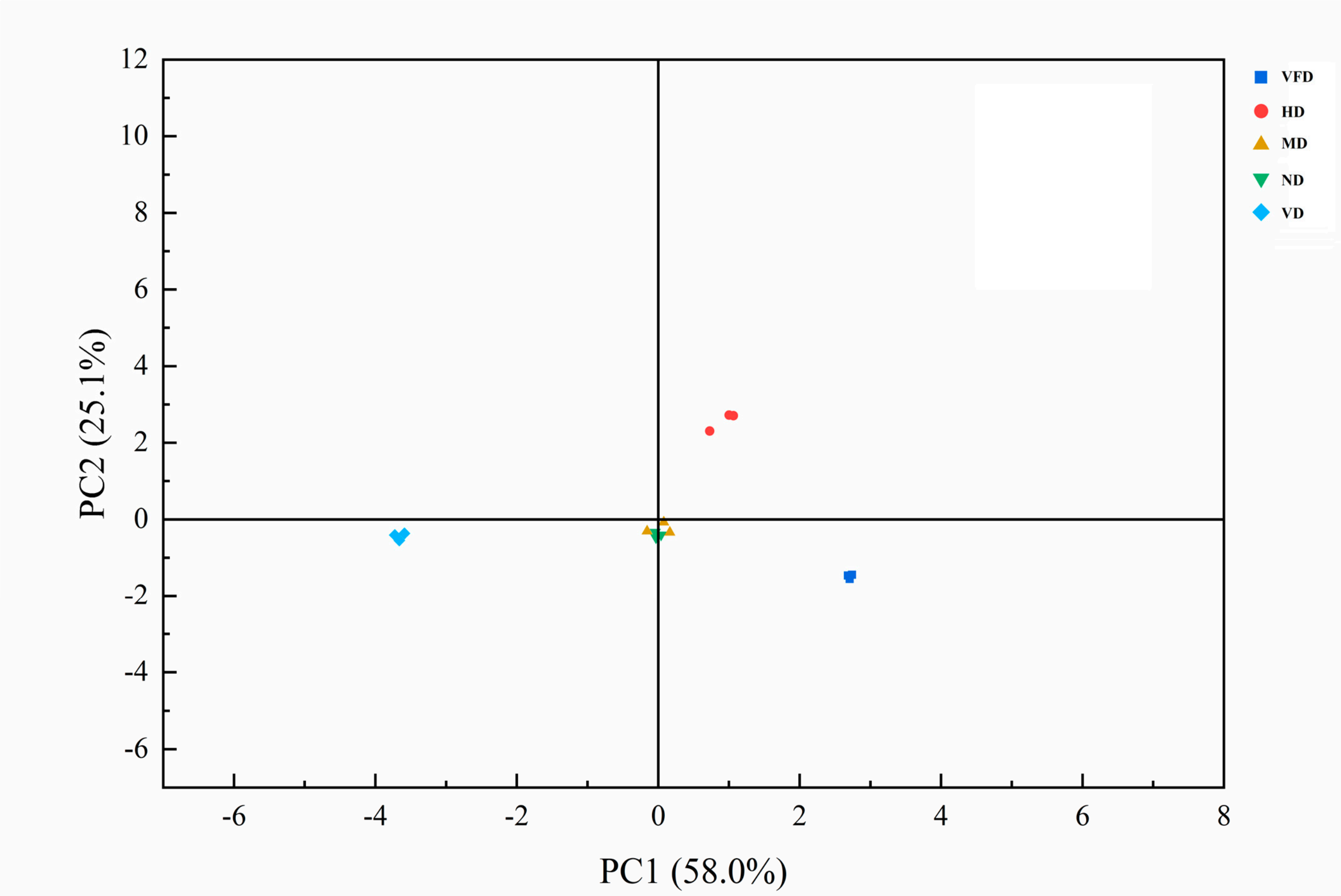

Figure S3. Principal components analysis of main active ingredients of A*S* fruit under five different drying methods.

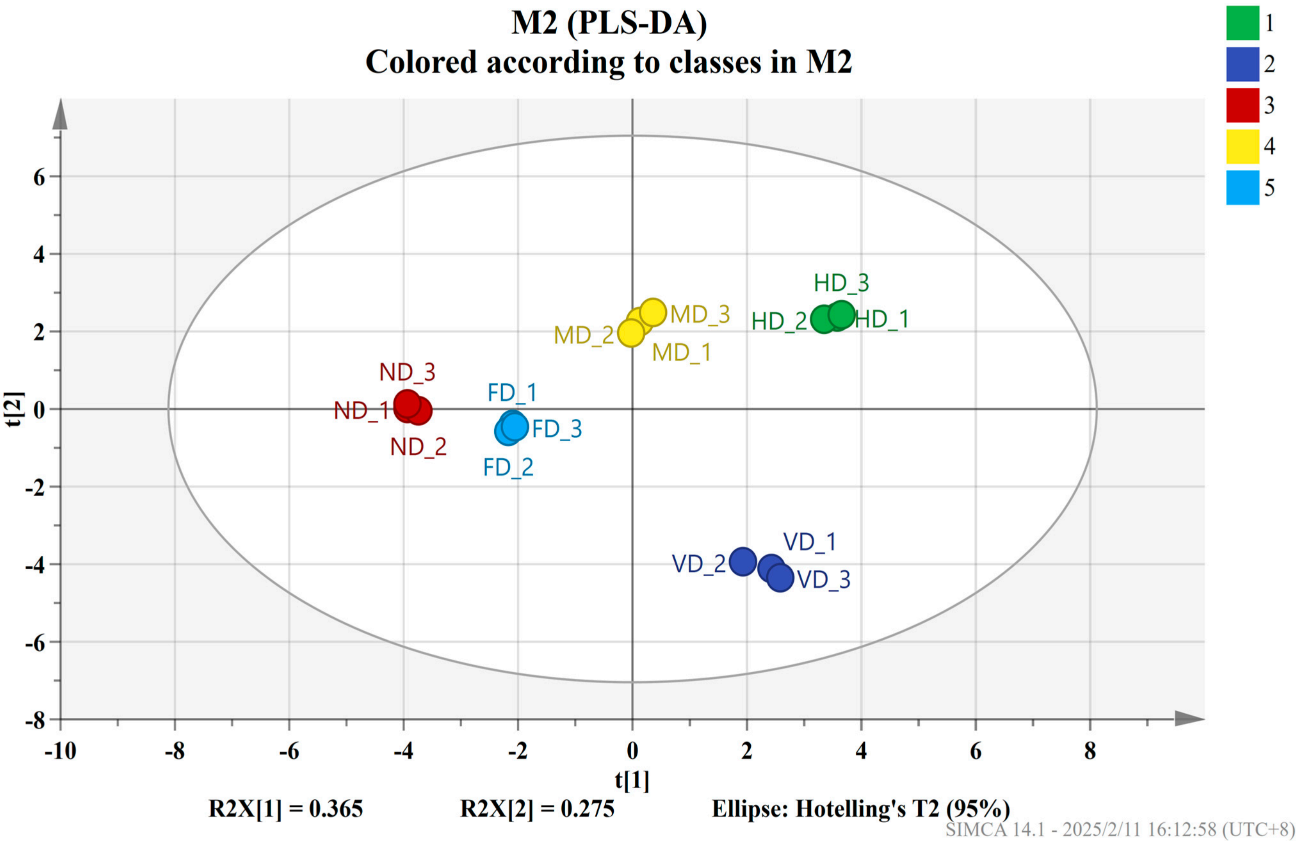

Figure S4. Partial Least Squares Discriminant Analysis of volatile compounds in AS fruit subjected to five different drying methods.

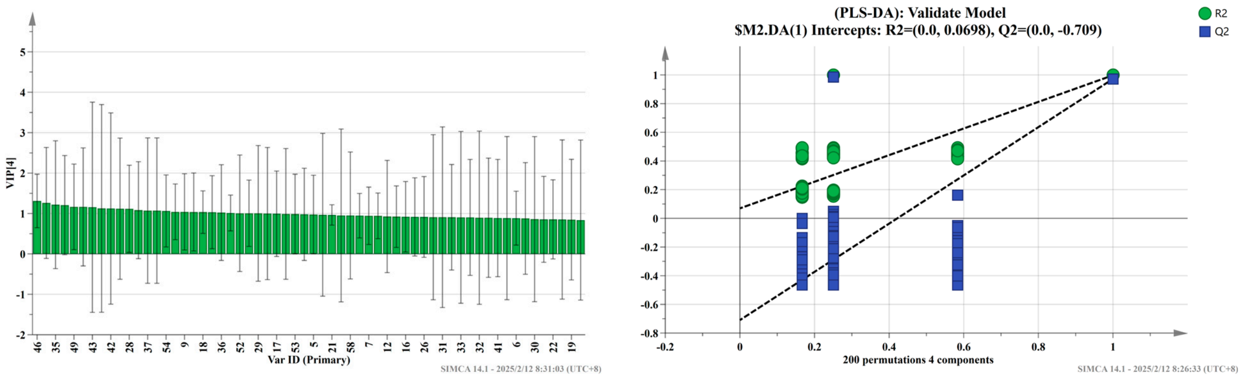

(**a**) (**b**)

Figure S5. Variable importance in projection (VIP) values of characteristic volatile compounds **(a)** and permutation test results **(b)** for volatile compounds in five groups of AS fruit samples subjected to different drying methods.
